# De novo antibody identification in human blood from full-length single B cell transcriptomics and matching haplotype-resolved germline assemblies

**DOI:** 10.1101/gr.279392.124

**Published:** 2025-04

**Authors:** John Beaulaurier, Lynn Ly, J. Andrew Duty, Carly Tyer, Christian Stevens, Chuan-Tien Hung, Akash Sookdeo, Alex W. Drong, Shreyas Kowdle, Axel Guzman-Solis, Domenico Tortorella, Daniel J. Turner, Sissel Juul, Scott Hickey, Benhur Lee

**Affiliations:** 1 Oxford Nanopore Technologies, Inc., New York, New York 10013, USA;; 2 Icahn School of Medicine at Mount Sinai, New York, New York 10029, USA

## Abstract

Immunoglobulin (*IGH*, *IGK*, *IGL*) loci in the human genome are highly polymorphic regions that encode the building blocks of the light and heavy chain IG proteins that dimerize to form antibodies. The processes of V(D)J recombination and somatic hypermutation in B cells are responsible for creating an enormous reservoir of highly specific antibodies capable of binding a vast array of possible antigens. However, the antibody repertoire is fundamentally limited by the set of variable (V), diversity (D), and joining (J) alleles present in the germline IG loci. To better understand how the germline IG haplotypes contribute to the expressed antibody repertoire, we combined genome sequencing of the germline IG loci with single-cell transcriptome sequencing of B cells from the same donor. Sequencing and assembly of the germline IG loci captured the *IGH* locus in a single fully phased contig where the maternal and paternal contributions to the germline V, D, and J repertoire can be fully resolved. The B cells were collected following a measles, mumps, and rubella (MMR) vaccination, resulting in a population of cells that were activated in response to this specific immune challenge. Single-cell, full-length transcriptome sequencing of these B cells results in whole transcriptome characterization of each cell, as well as highly accurate consensus sequences for the somatically rearranged and hypermutated light and heavy chain IG transcripts. A subset of antibodies synthesized based on their consensus heavy and light chain transcript sequences demonstrate binding to measles antigens and neutralization of authentic measles virus.

The immune system is a complex network of cells, tissues, and organs that work together to defend the body against infections and foreign substances. It is broadly categorized into two main components: the innate immune system, which responds quickly to infections but does not exhibit specificity or memory, and the adaptive immune system, which provides a specific, targeted response to pathogens, and which develops a memory of past exposures. The adaptive immune system is composed of T cells, which create a cell-mediated response to pathogen exposure, and B cells, which produce antibodies. To recognize a wide variety of pathogens, both T and B cells must be able to produce highly diverse antigen receptors. This is achieved by extensive recombination of their receptor-gene segments, creating a unique immune repertoire in every individual (Tonegawa 1983; Watson et al. 2017). In B cell maturation, multiple distinct subtypes of cells arise during an immune response. These include plasma cells and plasmablasts, which are transient antibody-secreting populations appearing during the acute phase of an immune response, and memory B cells (MBCs), which produce low levels of antibodies in order to “remember” a specific antigen and can quickly differentiate into plasmablasts upon reexposure to a previously encountered antigen. As a result, reexposure to the same or closely related pathogen or antigen results in clonal expansion from the circulating memory B cell population, enabling a more rapid and robust immune response.

Sequencing immune-gene transcripts of individuals within a population reveals allelic diversity and allows comparison between immune responses to pathogenic antagonists, cancer, and autoimmune diseases (Boyd et al. 2010; Rodriguez et al. 2023; Hansen et al. 2024). Single-cell transcriptome sequencing of immune cells enables closer examination of heterogeneity within these cell populations and has led to a greater understanding of immune cell gene expression, heavy-light chain pairing, VDJ (variable, diversity, and joining) recombination, somatic hypermutation, and class-switch recombination than can be derived from bulk analyses (Dudley et al. 2005; Di Noia and Neuberger 2007; Papalexi and Satija 2018; Jaffe et al. 2022). Single-cell sequencing using long-read approaches can produce reads that span entire transcripts, removing the need for isoform reconstruction and is, therefore, preferable to short-read approaches that can miss novel transcripts and isoforms (Singh et al. 2019; Subas Satish et al. 2022; Engblom et al. 2023). This is particularly important in the context of antibody sequencing because the variable domain must be unambiguously paired with the constant domain in order to determine both the VDJ recombination and the class-switch recombination in the same antibody transcript. Long-read, single-cell transcriptomes of B cells paired with high-quality germline genome assembly enables unambiguous annotation of allele specific transcripts after recombination. Additionally, germline immunoglobulin (IG) assembly makes it possible to filter antibody clones based on the presence or absence of specific alleles, for example variable alleles, that are present within an individual versus ruling out closely matched alleles that are not present within the individual's germline sequence.

While single-cell methods have provided novel insights into the transcriptional and posttranscriptional processes involved in the immune response, the genetic components have been harder to unravel. This is due to both the high levels of polymorphism and the structural complexity of the loci involved (Watson et al. 2013; Rodriguez et al. 2020; Nurk et al. 2022; Rodriguez et al. 2023). The B cell receptor (BCR) is composed of IG heavy and light chains. The heavy chain is encoded in humans by the *IGH* locus on Chromosome 14 and the light chain is encoded by either the IG kappa (*IGK*) locus on Chromosome 2 or the IG lambda (*IGL*) locus on Chromosome 22. The IG loci have complex genomic organization, high sequence diversity and undergo extensive rearrangement in B cells during antigen-mediated activation and maturation. Consequently, assembly of these loci benefits from use of long reads that can resolve their complex structures and improve haplotype phasing (Kolmogorov et al. 2023). However, individual single-cell transcriptomes have so far not been paired with germline assemblies from the same donor. Haplotyped structural variants and heterogeneity of IG V, D, J, and C gene segments may affect the antibody repertoire of a given individual, representing the potential of future precision medicine based on individual variation (Kidd et al. 2016; Kenter et al. 2021; Peres et al. 2023).

In the work presented here, we generated full-length single-cell antibody transcripts from MBCs and antibody-secreting cells (ASCs), as well as haplotype-resolved germline assemblies of IG loci from a single donor (Fig. 1). The assemblies were annotated against IMGT/GENE-DB (Giudicelli et al. 2022), a public database of IG V, D, J, and C sequences, and the transcripts were mapped against this personalized set of IG genes. Our approach allows some allelic annotations to be filtered out if they are not present in the matched germline locus, as those reference transcripts could not have been translated into an antibody in that individual. Finally, we validated functional antibody clones against viral antigens and characterized the clonal diversity at the single-cell level of paired heavy and light chain clones.

**Figure 1.**
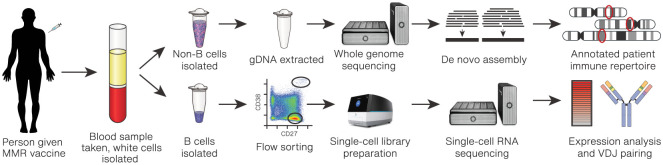
Experimental design schematic. Peripheral blood mononuclear cells (PBMCs) collected from a donor at 6 days post-MMR vaccination were separated into B cells for single-cell sequencing and monocytes for germline sequencing and assembly.

## Results

### A donor-specific IGH assembly is highly contiguous and fully phased

To assess the full repertoire of donor-specific germline immune alleles, we depleted B and T cells from whole blood and extracted DNA for whole genome nanopore sequencing (Methods). Approximately 45× coverage of nanopore sequencing reads was de novo assembled and phased, resulting in a highly contiguous personalized whole genome assembly (statistics in Supplemental Table S1). The *IGH*, *IGL*, and *IGK* loci were isolated from this assembly for in-depth annotation and study.

**Figure 2.**
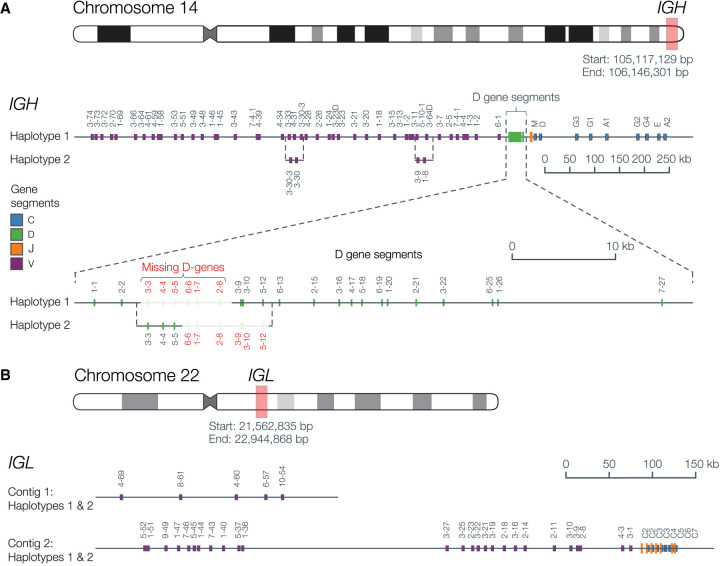
A donor-specific, haploid assembly was created using Flye and HapDup (Methods). Colored bars indicate the position on the contig of functional V, D, J, and C gene segments. (*A*) The fully phased *IGH* locus, with the dashed lines depicting alternative structures in sections where the assembly differs structurally between the two haplotypes, such as the *IGHV3-9* and *IGHV1-8* structural variant. The D gene cluster is expanded to show that a block of D gene segments is missing from each haplotype. (*B*) The *IGL* locus, where the *IGL* C V-CLUSTER was assembled into a separate contig from the remaining V-CLUSTERs and the J-C-CLUSTERs. All gene segments were shared between both haplotypes.

The 2 Mb *IGH* region was contained within a 79 Mb contig and separated into two phase blocks. The phase block gap did not contain heterozygous single-nucleotide polymorphisms (SNPs), but was manually resolvable using a heterozygous 172 bp deletion between *IGHV3-20* and the pseudogene *IGHV3-19*, resulting in a fully phased *IGH* region (Fig. 2A). Additionally, structural variant calling supported the inclusion of a 37 kb alternate contig representing the known 5-10-1/3-64D structural variant (Lefranc 2001a), which was manually placed into haplotype 2. All *IGHJ* and constant gene segments were present in the expected order. A block of *IGHD* gene segments was missing from each haplotype; haplotype 1 was missing D3-3 through D2-8, and haplotype 2 was missing D6-6 through D5-1.

The *IGL* V clusters A and B, as well as the J-C cluster, were contained on a fully phased 26 Mb contig; V cluster C was present on a separate phased contig (Fig. 2B). The *IGL* region typically contains 7–11 highly similar J-C cassettes, ranging from 3.5 to 5.6 kb, each containing a J and a C gene segment (Lefranc 2001c). This *IGL* J-C cluster contains nine cassettes, including one novel *IGLC3* allele that differed from the closest known alleles by a single SNV (Supplemental Fig. S1).

Although ∼660 kb of *IGK* sequence was assembled, it was relatively discontiguous (split into 7+ contigs). Furthermore, *IGK* could not be fully resolved due to two major challenges: (1) It consists of two highly similar ∼400 kb segmental duplications and (2) these segmental duplications are separated by 800 kb of highly repetitive sequence (Lefranc 2001b). Consequently, the assembler collapsed the homologous sequences between the two segmental duplications, and allele calling was not possible for collapsed genes.

Of the 295 total annotated IG gene segments, 288 (97.6%) matched perfectly with entries in the IMGT or OGRDB databases (Lees et al. 2020). We identified 2 *IGLV* alleles, 1 *IGHV* allele, and 1 *IGLC* allele (which appeared in both haplotypes) with novel polymorphisms that are not present in the IMGT or OGRDB databases. Each of these novel alleles in the assembly are supported by reads that align to each locus (Supplemental Fig. S1), as well as by expressed IG transcripts (Supplemental Table S2). Reads aligned to the remaining two gene segments indicate the likely presence of a 1 nt deletion error in the assembled sequence. Of the three bona fide novel V gene alleles, two are in framework region 1, while the third (IGLV2-14*03-like) involved a Glu (E) to Asp (D) change in residue 56 (Kabat numbering) in CDR2. The latter could potentially impact antibody reactivity or provide a different pathway for affinity maturation, as has been shown for other germline polymorphisms (Yuan et al. 2023).

We observed consistent 5-methylcytosine (5mC) hypomethylation at CpG sites downstream from *IGHV* genes, as well as around the D and J gene clusters (Supplemental Fig. S2). The significance of this observation is unclear, but it warrants further investigation in future studies.

### Single-cell transcripts and isoforms are differentially expressed among B cell subpopulations and unambiguously annotated using nanopore sequencing

To understand the expression of immune genes in response to an immune challenge, our donor received a measles, mumps, and rubella (MMR) vaccination followed by a longitudinal series of blood draws. Daily PBMC samples were monitored for the appearance of CD27^++^/CD38^++^ expressing ASCs by fluorescence-activated cell sorting (FACS) (Supplemental Fig. S3). This putative population of ASCs appeared on day 6 postvaccination, at which point we collected 80 mL of whole blood for B cell enrichment and sorting. We used a multiparameter FACS strategy (Methods), involving doublet exclusion, viability gating, and a T/monocyte/NK cell exclusion step to ensure a highly pure population of CD19^+^/IgM^−^ B cells for sorting into CD38^++^/CD27^++^ ASCs in the P7 gate (referred to henceforth as the ASC gate) and CD38^−^/CD27^+^ MBCs in the P8 gate (MBC gate). Approximately 1000 and 30,000 cells were collected from the ASC and MBC gates, respectively, and were subsequently processed for single-cell transcriptome sequencing.

To characterize single-cell expression within the B cell subpopulations, the single cell, whole transcriptome libraries of both the ASC gate and MBC gate were split in half and sequenced by both short-read and nanopore sequencing platforms. Single-cell cDNA sequences from the short-read and nanopore libraries of both the ASC and MBC gates were compared for cell barcode overlap and unique molecular identifier (UMI) counts per cell. UMI comparisons revealed a high degree of concordance between the two sequencing technologies in both the ASC gate (Supplemental Fig. S4) and MBC gate (Supplemental Fig. S5).

**Figure 3.**
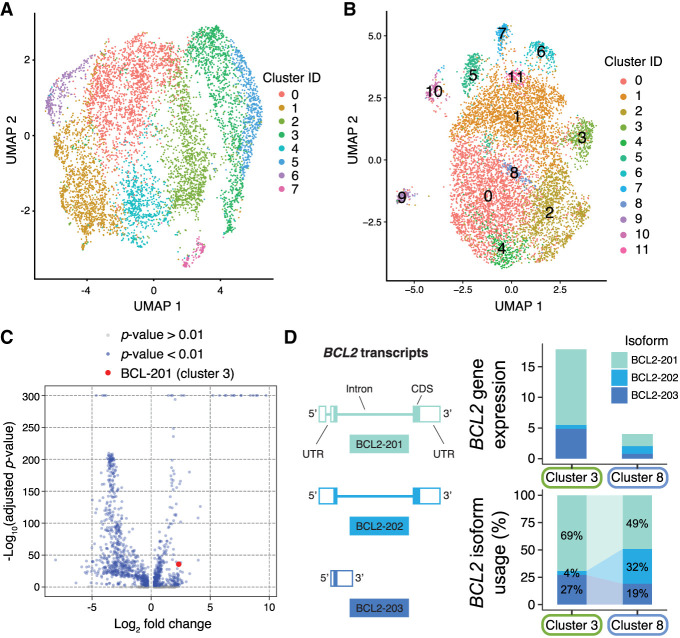
Single-cell expression data from the MBC gate. (*A*) Uniform Manifold Approximation and Projection (UMAP) visualization of cells clustered by gene expression quantified by short-read sequencing. (*B*) UMAP visualization of cells clustered by isoform expression quantified by nanopore sequencing. (*C*) Volcano plot showing isoforms that are differentially expressed in one cluster relative to the entire population of cells, with Bonferroni-adjusted *P*-values. One differentially expressed isoform of the apoptosis regulating gene *BCL2* is highlighted. (*D*) Cluster-specific expression of the *BCL2* gene and the differential transcript usage of three *BCL2* isoforms. BCL2–202 isoforms make up only 4% of *BCL2* transcripts in cluster 3 but represent 32% of *BCL2* transcripts in cluster 8.

Because nanopore sequencing generates reads covering full-length transcripts, expression was tabulated not only at the gene-level, but also at the transcript-level in order to quantify isoform expression of each gene. While the gene expression landscape of cells in the MBC gate can be assessed using short reads (Fig. 3A), the transcript-level expression provided by long-read sequencing provides an alternative view (Fig. 3B) with the potential for uncovering cell state differences that would be obscured at the level of gene expression. An analysis of differential transcript expression across cell clusters (Methods) revealed a total of 1785 transcripts whose expression in cells from one cluster was significantly higher or lower than in all cells in the remaining clusters (Fig. 3C; Supplemental Table S3).

The differentially expressed transcripts can highlight how different isoforms of the same gene can be preferentially expressed in some cells. For example, the apoptosis regulator *BCL2* has multiple splice variants (Warren et al. 2019) and shows distinct isoform usage patterns between clusters. In cluster 3, BCL2–201 dominates, with 69% of total *BCL2* expression, while BCL2–202 contributes only 4%. Cluster 8 shows lower overall *BCL2* expression but increased BCL2–202 usage at 32% (Fig. 3D). While both isoforms encode the same protein and differ only in their 5′ UTRs, this dramatic shift in isoform preference suggests important biological processes that would be missed by gene-level analysis alone.

**Figure 4.**
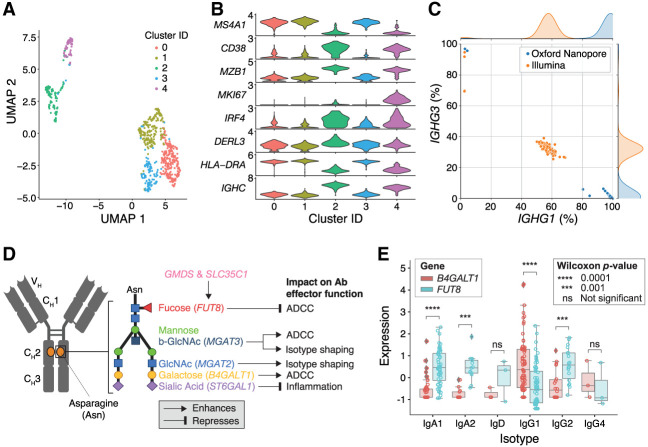
Single-cell expression data from ASC gate. (*A*) UMAP visualization of cells clustered by gene expression quantified by nanopore sequencing. (*B*) Expression of marker genes differentiating in each cluster. (*C*) Proportion of reads assigned to *IGHG1* and *IGHG3* within each cell from the ASC gate in both the short-read and nanopore sequencing data sets. (*D*) Schematic illustrating the genes involved in glycosylation of the conserved asparagine residue in the CH2 domain of the heavy chain. (*E*) Differential expression of two genes in the glycosylation pathway, *B4GALT1* and *FUT8*, in certain antibody isotypes of plasmablasts and plasma cells from the ASC gate. Each point represents a single cell.

For the ASC gate, gene expression clustering of the cells based on nanopore sequencing revealed multiple cellular subpopulations, including CD38^++^ plasmablasts and plasma cells (Fig. 4A). Due to dynamic gating during the sorting process, some CD27^+^/CD38^+^ activated MBCs were also collected in the ASC gate, but these are easily identifiable as a separate population due to their distinct expression profiles (Fig. 4A,B). Based on expression, cluster 2 appears to represent a population of nonproliferating plasma cells (*MKI67*-low, *IRF4*-high), while cells in cluster 4 are likely actively proliferating plasmablasts (*MKI67*-high, *IRF4*-low) (Fig 4B). Cells in both clusters 2 and 4 produce a high level of *IGHC* transcripts, consistent with their role in high production and secretion of antibodies. The remaining 374 CD38^−^ cells in clusters 0, 1, and 3 are most likely residual MBCs that were missorted into the ASC gate, as they have with low expression of *IGHC*, *DERL3*, *IRF4*, and *MKI67* but high expression of *MS4A1*/*CD20* and *HLA-DR*. Nonetheless, our expression data were clearly able to differentiate these cells from plasmablasts or plasma cells.

Antibody specificity derives from the sequence of the complementarity determining regions (CDRs) of the light and heavy chains. The isotype of an antibody, such as IgA, IgD, IgE, IgG, and IgM, is determined by the sequence of the constant domains in the heavy chain. IgG and IgA are further divided into subtypes based on the hinge region. While each cell is expected to produce antibodies of a single isotype and subtype (e.g., *IGHG1*), the short-read sequencing data revealed ambiguous isotype assignments. Most ASCs had a mix of *IGHG1* and *IGHG3* assignments from short reads, likely due to high sequence similarity in the limited portion of the constant domains that are captured in the fragmented short-read sequencing library. In contrast, the nanopore sequencing data showed no such ambiguous isotype and subtype assignments, with nearly all full-length transcript reads from each cell mapping definitively to either *IGHG1* or *IGHG3* (Fig. 4C).

### Antibody glycosylation phenotypes

Differential glycosylation of antibodies affects their Fc effector functions, serum half-life, and isotype shaping (Irvine and Alter 2020). GWAS studies have also linked SNPs in glycosyltransferases, such as *ST6GAL1*, *B4GALT1*, *FUT8*, and *MGAT3*, to IgG *N*-glycosylation phenotypes and certain isotypes (Shen et al. 2017; Wahl et al. 2018). Several core glycosylation genes are known to have specific impact on antibody-dependent cellular cytotoxicity (ADCC), isotype shaping, and inflammation (Fig. 4D). However, it is unclear if these genes are preferentially expressed at the level of single B cell clones.

We examined the expression of core antibody glycosylation genes in both the ASC and MBC gates. In the majority of plasmablasts and plasma cells in the ASC gate, *FUT8*, a fucosyltransferase, was significantly overexpressed relative to *B4GALT1* in cells producing IgA1 and IgA2 (Fig. 4E). The reverse was true in cells producing IgG1. Since increased fucosylation is negatively correlated with ADCC function, our data suggest attenuation of ADCC activity in the secreted IgAs and enhanced ADCC activity in the secreted IgGs.

In the MBC gate, glycosylation patterns were not broadly associated with specific isotypes. However, high expression of *MGAT3*, *MGAT2*, and *SLC35C1* was restricted to a minority of MBCs (Supplemental Fig. S6). Furthermore, high *MGAT3* and *SLC35C1* expression in these MBC clones was mutually exclusive, consistent with their opposing roles in modulating ADCC activity.

### IG transcript enrichment and germline assembly mapping enables high-confidence consensus sequences for antibody synthesis

**Figure 5.**
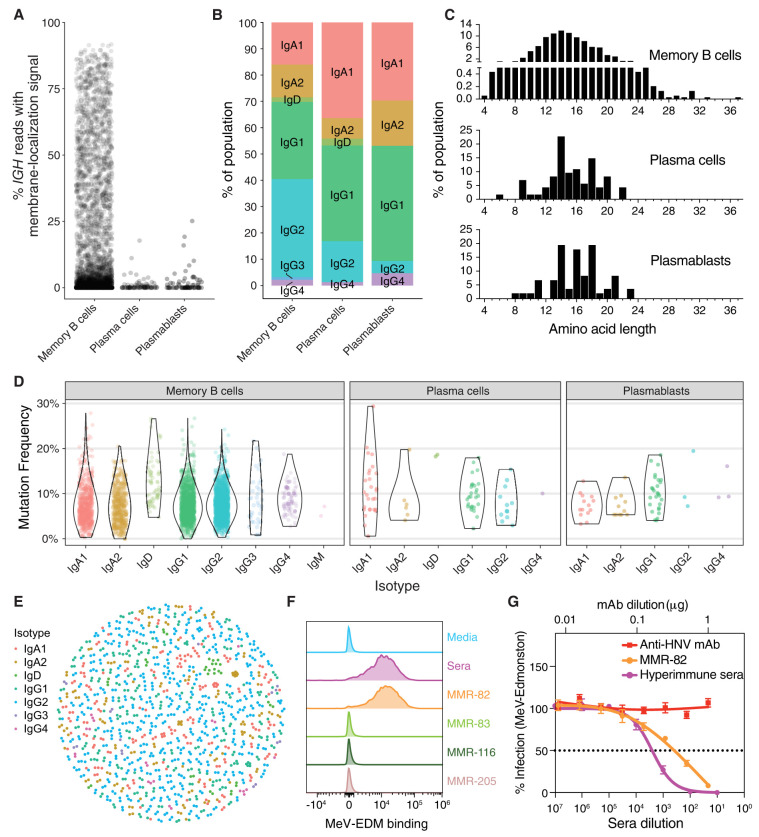
Characterizing single-cell IG sequences and antibody synthesis. (*A*) The percentage of *IGH* reads in each cell possessing the M exons indicative of a membrane-bound antibody, stratified by cell type. (*B*) Proportions of each isotype identified from consensus *IGH* sequences, stratified by cell type. (*C*) CDR3 amino acid length distributions for all productive *IGH* consensus sequences, stratified by cell type. CDR3 lengths are enumerated between the junction anchor residues: Cys 104 and Try 118 (IMGT numbering) for each sequence. (*D*) Mutation frequency compared to the germline sequences in the FWR1–4 and CDR1–3 regions for each cell type and isotype. (*E*) Dandelion plot of sequences clustered within each nonsingleton clone by VDJ amino acid sequence similarity. (*F*) Putative positive recombinantly expressed antibodies sequenced from the ASC gate were screened by flow cytometry for binding to MeV surface expressed proteins (F or RBP/H) on virus infected Raji-DC-SIGN B cells (multiplicity of infection [MOI] 0.01). MMR-vaccinated, hyper-immune sera from the donor at the time of cell collection was used as a positive control. (*G*) Neutralization assay in Vero cells for MMR-82 (MeV+) and hyper-immune sera against a GFP-expressing MeV-Edmonston recombinant virus (1000 IU/96-well). Data are presented as percent infection (mean ± SD) compared to infection in media alone (set as 100%). Experiments were performed in triplicate. Anti-HNV monoclonal antibody (mAb) is an irrelevant IgG1 mAb against an unrelated virus and serves a negative control.

To first quantify the relative proportions of secreted and membrane-bound antibodies in each cell, we searched for reads containing the *IGHC* M exons, which reside on the 3′ end of the *IGH* transcript and harbor the *trans*-membrane, connecting, and cytoplasmic tail regions. Although the majority of transcripts contained the secreted form of *IGHC* lacking the M exons, a subset of MBCs primarily expressed membrane-bound transcripts (Fig. 5A). In contrast, all of the ASCs primarily expressed the secreted forms.

Next, we paired heavy and light chain IG transcripts from each cell so that functional antibodies could be generated directly from the consensus sequences. The ASCs generated a large number of IG transcript reads from the whole transcriptome sequencing of each cell: on average 5319 from the light chain and 357 from the heavy chain.

The MBCs, however, had a much more diverse transcriptome that resulted in fewer IG transcript reads from each cell. To increase the IG transcript coverage for these cells, a second sequencing library was generated using biotin probe enrichment to enrich for *IGH*, *IGL*, and *IGK*-containing transcripts (Methods). This resulted in a 130-fold enrichment in the percentage of IG transcripts compared to the matching whole transcriptome sequencing data set and made it possible to recreate high-accuracy heavy and light chain sequences from several thousand MBCs.

We created a custom pipeline (Supplemental Fig. S7) to produce heavy and light chain IG consensus sequences for each cell (Methods). This pipeline generated 761 and 7653 consensus sequences that were predicted to be productive from 616 and 5564 cells in the ASC and MBC gates, respectively. We determined the isotype of each consensus sequence using IgBLAST (Fig. 5B). The length distributions varied by isotype and were consistent with expectations based on the literature and reference database (IMGT–GENE-DB) (Supplemental Fig. S8; Lefranc et al. 2005). To assess the accuracy of these consensus sequences, we examined the CH1 regions from the constant genes because they are unaffected by somatic hypermutation. In the 4040 productive *IGH* consensus sequences, we found 96% of the CH1 regions to be identical to the germline CH1 alleles, with a mean accuracy of 99.984% (Supplemental Fig. S9).

Observed variation in the length of sequences from each isotype can be explained by the variable lengths of the CDR3 sequences for each subpopulation (Fig. 5C). A Gaussian-like distribution of CDR3 lengths in the memory population was consistent with average CDR3 lengths in peripheral MBC populations in healthy individuals, with a median of 14 amino acids (aa) (Tian et al. 2007; Duty et al. 2009; Dong et al. 2020). We observed that 0.2% (eight sequences) of the MBC contained CDR3 lengths of 30 aa or longer, including one CDR3 of 37 aa, that were not selected in the ASC populations. Nonsymmetrical distributions in the ASC populations, plasma cells, and plasmablasts show evidence of length biases, indicative of selection.

Nucleotide mutation frequencies observed in the V gene segment were within the standard range for postgerminal center, class-switched B cell populations with an average mutation frequency of 7.7% across all isotypes (Fig. 5D; Wrammert et al. 2008; Dong et al. 2020; Budeus et al. 2023). Analysis of mutation by isotype revealed rarer IgD MBC and plasma cell populations (1% and 0.03% abundance within the two populations, respectively), which did not contain IgM transcripts, had high mutation frequencies (>12%), and had increased JH6 usage (see below section on germline-specific repertoire analysis). This population is reminiscent of class-switched IgD B cell populations, mostly observed in germinal centers, that are reported to have high mutation rates (>10%) and preferential usage of JH6 segments (Zheng et al. 2004; Koelsch et al. 2007; Seifert and Küppers 2016; Budeus et al. 2023).

The Immcantation (Gupta et al. 2015) suite of tools was used to call clones based on the heavy chain junction length and sequence identity; subclones were called by light chains when available; 1219 clones had multiple members with cluster sizes ranging from 2 to 16 (Fig. 5E). Certain clones varied in their heterogeneity; for example, a group containing 14 IgD clones had more divergent sequences than a more tightly clustered group of 11 IgA2 clones.

Because the sequencing data were generated using Oxford Nanopore Technologies’ R9 flow cells, we assessed whether significant consensus accuracy improvements could be expected using the newer R10 flow cells. We sequenced leftover cDNA from the MBC gate using an R10 flow cell and compared the resulting consensus VDJ regions to the cell barcode- and IG locus-matched sequences from the R9 data. Overall, the proportion of productive consensus sequences increased from 78% to 87%. The jump is mainly driven by *IGH* productivity, which increased from 80% to 95%; 656 out of 1010 barcodes with unproductive *IGH* sequences in the R9 data had productive *IGH* sequences in the R10 data, with the vast majority becoming productive due to a G homopolymer correction. Still, among the filtered sequences used for the final R9 data set, the vast majority (94%) of sequences agreed with the R10 consensus. For future experiments, R10 would give a higher proportion of productive data, but R9 data are still useful with sufficient filtering.

### Antibody synthesis and binding results

To investigate the function of novel antibody sequences identified in the ASCs, we generated 100 monoclonal antibodies based on consensus heavy and light chain sequences. The first batch of 50 antibodies was synthesized as full-length IgG1 IGs, and the subsequent 50 were engineered as single-chain variable fragment (scFv)-IgG1-Fc fusions. This approach allowed for both the assessment of native antibody binding characteristics (full-length IgG1) and the rapid screening of multiple antibody specificities simultaneously (see Methods).

Initially, the first batch of antibodies were screened for binding to MMR virus antigens via commercial ELISA kits. One of the full-length IgG1 antibodies, MMR-32 yielded positive binding results. MMR-32 displayed apparent polyreactivity as it bound to ELISA plates coated with both mumps and rubella virus antigens (Supplemental Fig. S10A–C). None of these antibodies exhibited neutralizing activity against MeV, MuV, or RubV (Supplemental Fig. S10D–F).

Subsequent screening of the 50 scFv-Fc fusions by FACS on ExpiCHO cells transiently transfected with MeV and MuV fusion (F) and receptor-binding protein (RBP) expression constructs identified several clones demonstrating binding to MeV-F proteins (Supplemental Fig. S11), notably MMR-82 and MMR-205. Further characterization confirmed MMR-82 binding to native MeV envelope proteins on infected Raji-B cells (Fig. 5F). Importantly, MMR-82 effectively neutralized MeV infection (Fig. 5G).

Since these ASCs were not antigen-baited, we tested whether any of the recovered antibodies were specific to other common infectious agents. Since up to 85% of adults >40 years are seropositive for human cytomegalovirus (HCMV), we screened 50 mAbs for reactivity against HCMV in an immunofluorescent assay and detected at least one positive clone (MMR-8) (Supplemental Fig. S12).

None of the antibodies, including the scFv-Fc fusions, demonstrated positive binding to the viral antigens in this ELISA format while hyper-immune polyclonal sera demonstrated the expected reactivity in the ELISA assays (Supplemental Fig. S10A–C). The lack of reactivity among the monoclonal antibodies highlights the possibility of false-negative results with ELISA-based screening methods since MMR-82 was obviously a positive binder to MeV-F.

### Germline-specific repertoire analysis of antibody sequences confirm B cell class and allows haplotype distinction

**Figure 6.**
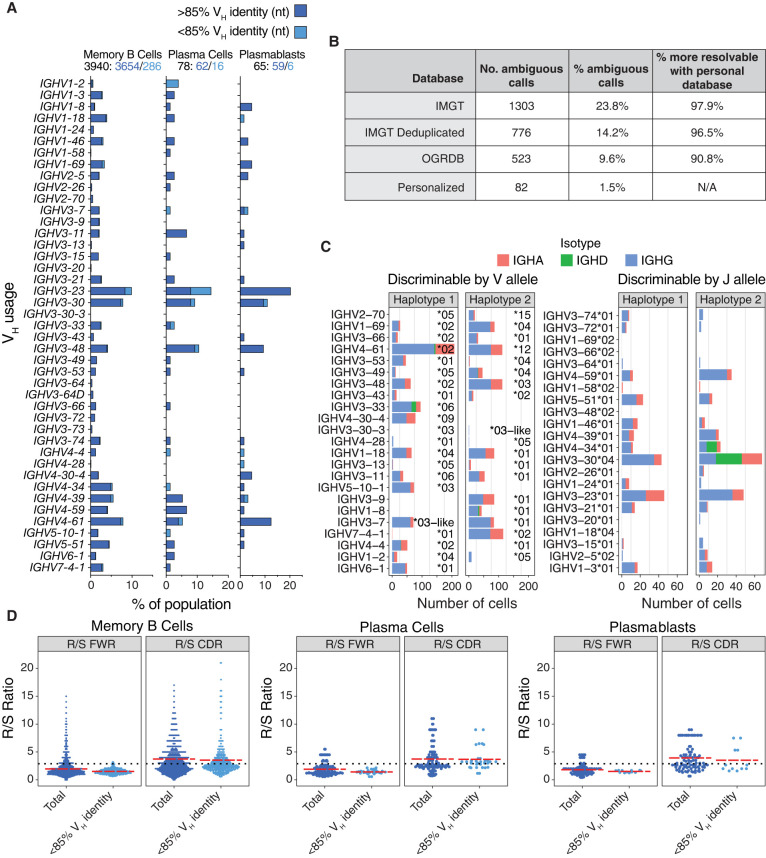
Donor-specific V gene usage and mutation rates. (*A*) Overall IGHV gene usage frequency for all complete and productive heavy chain consensus sequences with an in-frame CDR3. Cells with a low V gene identity, high mutation rates (≥15%) are stratified (light blue), as they are sometimes filtered out by programs such as Hi-V-QUEST (IMGT). (*B*) Statistics for ambiguous *IGH* and *IGL* V gene calls when using IgBLAST with various databases. (*C*) V gene usage for cells expressing one of the heterozygous V alleles (*left*), or that expressed a homozygous V allele but were haplotype-discriminable by a heterozygous J allele (*right*). (*D*) Ratio of replacement to silent mutations (R/S) in framework regions (FWRs) versus CDRs. The dotted line represents theoretical R/S ratios for unselected CDRs at R/S = 2.9. The red lines are the mean R/S values. The elevated replacement mutations in the CDRs suggest cells that have undergone antigen selection.

Our pipeline enabled unambiguous assignment of *IGH* and *IGL* gene family usage (Fig. 6A; Supplemental Figs. S13, S14), revealing distinct gene segment repertoires in cells collected from the MBC and ASC gates. Similarly, *IGK* repertoires, assigned using the IMGT–GENE reference database, showed distinct VK segment usage across populations (Supplemental Fig. S14). The MBCs exhibited a diverse VH and JH segment repertoire, with preferential use of VH3 and VH4 gene families (Fig. 6A), and JH4 segments (Supplemental Fig. S14), typical of healthy adult MBCs (Tian et al. 2007). IgD-only MBCs expressed a preferential use of JH6 segments reminiscent of IgD class-switched B cells (63.89% JH6 usage vs. 19.1% in total memory cells vs. 20.9% in IgG1 memory cells) (Supplemental Fig. S15). In contrast, plasmablasts and plasma cells displayed a more restricted and biased VH repertoire, suggesting selective biases for antigen-specific ASCs (Fig. 6A).

A subset of sequences showed low V segment identity (<85%) against germline alleles, with mutation rates of 7%–9% for MBCs and plasmablasts, and 20% for plasma cells. Though 11% in MBCs and 13% in plasma cells were from the IgD-only population, most of these highly mutated sequences were on IgG and IgA bearing cells. Some highly mutated B cells are to be expected in healthy adults, as multiple exposures to the same immunizing antigens can recall B cells into further maturation rounds. We found population-specific VH usage biases in these highly mutated sequences (Fig. 6A, light blue bars). For example, VH1–2 segments were exclusive to the plasma cell population, suggesting that these patterns reflect B cell maturation rather than technical artifacts.

When using a deduplicated IMGT–GENE database with IgBLAST 14% of all functional V allele calls representing 57 different *IGH* and *IGL* genes are ambiguously assigned to multiple equally likely alleles. When using a personalized reference database, ambiguous calls are reduced by over 90%; only 1.5% of calls, representing 15 genes, remain ambiguous (Fig. 6B). For example, while *IGLV10-54* allele assignments remain ambiguous even when using the personalized database, the allelic ambiguity for *IGHV3-30* is completely resolved by using the personalized database (Supplemental Fig. S16). The unambiguous calls are also needed to distinguish transcripts from each haplotype.

Sequences can be traced back to a specific haplotype if they include a gene or allele that is only present on one haplotype. Fifty-five percent of *IGH* consensus sequences could be assigned a haplotype based on a heterozygous *IGHV* or *IGHJ* allele; 27% and 28% of consensus sequences came from haplotypes 1 and 2, respectively. Only 0.3% of the consensus sequences had a contradictory haplotype assignment between the V allele and the J allele, which supports the phasing accuracy of the assembly and is likely the result of hypermutation. Although the overall usage of both haplotypes is similar, certain alleles are used more often than others. For example, IGHV7-4-1*02 was used by 115 cells, versus a single cell using the *01 allele (Fig. 6C). The latter is known to be a poorly expressed VH allele in humans (Ohlin 2021). Two other poorly expressed human *IGHV* alleles (IGHV1-2*05 and IGHV4-4*01), known to be in linkage disequilibrium and typically found on the same haplotype (Ohlin 2021), were also present in our haplotype 2 and were found in 9 and 0 cells, respectively (Fig. 6C). Altogether, these data support the veracity of our analytic pipeline.

Mutational analysis of *IGH* shows an elevated ratio of replacement to silent mutations (R/S) in the CDR regions (mean = 3.7–3.9 depending on cell type) compared to the FWR and compared to an estimated theoretical R/S ratio previously calculated for unselected, random mutation (2.9) (Fig. 6D; Dörner et al. 1997, Wrammert et al. 2008; Cho et al. 2019). Increases in average R/S ratios of CDRs from multiple rounds of affinity maturation and selection in germinal center reactions is generally observed in memory and ASC B cell populations, and similarly, we see these increased averages for our selected populations. The R/S pattern holds true for the consensus sequences regardless of the level of *IGHV* nucleotide identity.

## Discussion

Our study shows that nanopore full-length, single-cell transcriptome sequencing can be used to explore multiple important features of the reactive antibody repertoire. We found that active ASCs like the plasmablasts and plasma cells found in the CD27^++^/CD38^++^ ASC gate expressed a sufficient number of IG heavy and light chain transcripts to enable the creation of high-quality, full-length consensus sequences without the need for any enrichment approaches that would bias the expression levels observed in each cell. Importantly, these consensus sequences captured not just the V(D)J domains, but also the complete C gene segments (and thus the antibody isotype), as well as the 3′ M exons that determine whether the antibody is destined to be secreted or trafficked to the cell membrane. Unambiguous isotype assignments are crucial for understanding the downstream effector function of each antibody and its role in the larger response to an immune challenge.

The consensus sequences obtained from the MBCs revealed an antigen-experienced repertoire that reflected the diversity of single antibodies and only a modest number of clonal expansions. We surveyed the landscape of antibody features across thousands of MBCs, including isotype, V gene usage, CDR3 lengths, and mutation rates. We observed V gene usage frequencies commonly seen in repertoires of post germinal center, isotype switched B cells (Tian et al. 2007; Glanville et al. 2009, 2011). V gene repertoire was also influenced by selection, as we observed differing patterns of V segment usage between the memory and antibody-secreting populations, implying positive selection of antibody-producing B cells with favorable antigen binding attributes.

Sequencing of the full-length IG transcripts permitted a complete evaluation of the full spectrum of CDR3 lengths (between 4 and 37 aa) in our collected antibody repertoire. We were able to capture a rare few with CDR3 lengths over 30 aa; long CDR3 antibodies, while often rare due to increased autoreactive potential (Wardemann et al. 2003; Larimore et al. 2012; Georgiou et al. 2014), have significantly been associated with a variety of protective traits in infectious disease, including associations with highly potent, broadly neutralizing antibody responses developed in patients with chronic infections like HIV (Yu and Guan 2014). These findings should be considered when choosing a sequencing platform for future antibody repertoire studies.

A matching whole transcriptome library from the MBC gate revealed preferential isoform expression in certain clusters of MBCs, indicating a level of heterogeneity within the population that is hidden when viewed at the level of gene expression. While we highlight one example of the critical apoptosis regulator *BCL2*, future studies will undoubtedly reveal numerous associations and mechanisms driven by isoform-level, rather than gene-level, expression patterns in single B cells.

The differential expression of two key glycosylation pathway genes, *B4GALT1* (IgG1) and *FUT8* (IgA1/IgA2), within specific antibody isotypes (Fig. 4D,E) unveils a layer of functional specialization that may define the fine-tuning of immune responses. For instance, *B4GALT1* is instrumental in the addition of GlcNAc moieties to *N*-glycans, while *FUT8* is responsible for adding fucose residues to the basal GlcNAc core, modifications known to increase and dampen ADCC activity, respectively (Wang and Ravetch 2019; Nimmerjahn and Werner 2021). To our knowledge, this is the first report to associate expression of effector function modifying *N*-glycan genes to B cell clones at a single-cell level. Furthermore, the mutually exclusive expression of *MGAT3* and *SLC35C1* in certain MBCs highlights their opposing roles in regulating ADCC activity. The nuanced interplay between these glycosylation patterns and specific antibody isotypes could be pivotal in orchestrating immune responses, where subtleties in effector function can have profound implications for pathogen neutralization, autoimmunity, and therapeutic antibody design (Wang 2019; Archer et al. 2022). Given the personalized nature of the immune response, our observations underscore the potential of leveraging single-cell sequencing technologies to dissect the molecular underpinnings of antibody function in the future of precision medicine.

We successfully generated and tested ∼100 antibody sequences, confirming both their accuracy and viability as IgG1 or scFv-Fc fusions. Among these, we identified MMR-82 as a high-affinity measles binder and neutralizer (Fig. 5F,G), along with MMR-32 and MMR-205 as lower affinity or polyreactive binders to cell surface antigens. In the absence of antigen baiting, it is unclear what the “hit-rate” should be as this has not been attempted before. In a study of MMR seropositive volunteers who received a booster shot (similar to our subject), low frequencies of virus-specific ASCs were found 1 week postvaccination; the median number of virus-specific ASCs per 10^6^ PBMCs were just 0.38 for mumps, 0.125 for measles, and 2.25 for rubella (Latner et al. 2011). Furthermore, only 62%, 43%, and 56% of individuals were positive for mumps, measles and rubella-specific ASCs. Assuming the median virus-specific ASC numbers are per 10^5^ B cells (10% PBMCs) and we sorted about 700,000 live B cells (Supplemental Fig. S3), obtaining only one virus-specific clone is within the expected range. The discovery of an anti-HCMV clone within our limited screen of just 50 mAb clones suggests that our pipeline can identify bona fide antibody sequences. The cognate antigens recognized by the remaining antibody clones are a subject for future studies.

Our focus in this study was to describe the antibody repertoire using a combination of single-cell, full-length transcriptomics, and a haplotyped long-read assembly of the *IGH* and *IGL* loci. Bulk adaptive immune receptor repertoire (AIRR-seq) (Ford et al. 2023) data have been paired with matching *IGH* genome assemblies before, but those genomes were generated from targeted sequencing approaches, which result in shorter reads and more discontiguous assemblies (mean # of contigs >100) (Rodriguez et al. 2023). In contrast, our donor's *IGH* locus was assembled into a single contig and phase block, showing that highly contiguous and fully phased *IGH* assemblies can be created and reliably annotated from standard long-read whole genome sequencing data.

We were able to successfully map the antibody repertoire from the plasmablasts, plasma cells, and MBCs to their cognate germline sequences, often in a haplotype-specific fashion where heterozygous V or J alleles permitted, with less V gene allele ambiguity than if relying on public databases for IgBLAST (1.5% ambiguous calls vs. 9.6%–23.8%). Our comprehensive list of alleles in both haplotypes reveals some that rarely or never produce productive transcripts, such as IGHV2-70*05.

Strategies similar to what has been described in this work may be useful for large-scale immunogenomic population studies and pangenome references covering a broad spectrum of IG allelic diversity. Larger data sets where single-cell B cell sequencing is paired with germline assembly will be critical for understanding haplotype-specific expression patterns for specific alleles, allelic silencing and imbalances, somatic hypermutation, and heavy-light chain pairing within individual B cells during cellular development.

## Methods

### Vaccination and study design

A combination MMR vaccine (M-M-R II, Merck and Co.) was administered subcutaneously to a consented donor. Six days postvaccination, 60 mL of whole blood was collected directly into BD Vacutainer CPT tubes (Becton Dickinson) and PBMCs were isolated by centrifugation at 1500*g* for 20 min at room temperature according to the manufacturer's instructions. The buffy coat containing PBMCs was removed, washed twice with 1× PBS and counted by hemocytometer.

### B cell enrichment and FACS

B cells were isolated using the Miltenyi B cell Isolation Kit II for negative B cell enrichment (Miltenyi Biotech, Auburn, CA) and passed over a Miltenyi LS column on a QuadraMACS magnetic separator with FACS buffer (1× PBS, 0.5% BSA, and 2 mM EDTA) according to manufacturer instructions. The collected flowthrough, containing untouched, enriched B cells, was washed twice in FACS Buffer and counted by hemocytometer with trypan blue staining (0.4% Trypan Blue in PBS solution) at a 1:10 dilution. The non-B cell fragment magnetically adsorbed to the column was also collected, counted, and resuspended in FACS buffer.

Enriched B cells were resuspended in ice cold FACs buffer at a density of 10^6^ cells/100 µL and treated with Human Fc Block (BD) according to manufacturer instructions. After blocking, the fraction was stained with an ASC/MBC-specific cocktail for FACS sorting (Supplemental Methods S1). After staining, cells were washed and resuspended in 1 mL 1× PBS and treated with LIVE/DEAD Fixable Near-IR Dead Cell Staining reagents (Thermo Fisher Scientific) according to manufacturer instruction. After live/dead staining cells were washed 3× in FACS buffer and resuspended in 200 µL FACS buffer for acquisition on a five laser BD FACS Aria II. Right before sorter acquisition, cells were strained through a 35 µm snap strainer (Thermo Fisher Scientific) to dislodge any large cell–cell adhesion events. B cells were separated from non-B cells by gating on the AF488/FITC negative gate against the FITC positive “non-B cell dump gate” separating CD3, CD4, and CD8 T cells and CD16 NK cells and monocytes. B cells were further enriched by CD19^+^ gating and subgated for CD27 and CD38 expression. ASCs (ASC gate, CD27^++^/CD38^++^) were separated from resting B memory cells (CD27^+^/CD38^−^) based on CD27 and CD38 expression.

### DNA extraction and germline whole genome sequencing

Genomic DNA was extracted from the monocyte cellular fraction using the Puregene Cell Kit (Qiagen) according to the modified Oxford Nanopore Technologies protocol available here: https://community.nanoporetech.com/extraction_methods/cell-line-dna-pure. Extracted DNA was further purified by isopropanol precipitation, quantified using a Qubit fluorometer (Thermo Fisher Scientific), and prepared for whole genome sequencing following the Ligation Sequencing Kit protocol (Oxford Nanopore Technologies SQK-LSK114) with long fragment buffer. Libraries were sequenced on a PromethION (Oxford Nanopore Technologies) with R10.4.1 flow cells and basecalled using Dorado duplex with R10 v4.2.0 super accuracy, CpG-aware models (Supplemental Table S4).

### Germline immune repertoire annotation

Genomic DNA was assembled into a haploid assembly using Flye (Kolmogorov et al. 2019), then converted into a diploid assembly with HapDup (Kolmogorov et al. 2023). Contigs were mapped to GRCh38 using minimap2 (Li 2018) to identify IG regions. Structural variants were called with Sniffles2 (Smolka et al. 2024) to validate assembly accuracy and incorporate known alternative haplotypes. The assembly was annotated by aligning V, D, J, and C alleles from IMGT–GENE-DB (Giudicelli et al. 2005) using BLASTN (Camacho et al. 2009), with custom parameters for each gene segment type. Custom BLAST databases were created using the annotated assembly's *IGH* and *IGL* alleles, combined with reference C genes and *IGK* alleles from IMGT–GENE-DB. Methylation frequency at each CpG was aggregated using Modkit pileup. Additional details are provided in Supplemental Methods S2.

### 10x Chromium processing

Sorted B cells in the ASC and MBC gates were separately processed for single-cell RNA-seq using the Chromium Next GEM Single-Cell 3′ Reagent kits v3.1 (10x Genomics) according to the manufacturer instructions with modifications: After the GEM-RT cleanup and full-length cDNA amplification step, 50% of the cDNA samples from each sorted cell population were saved for long-read cDNA sequencing. The other 50% were processed for 3′ gene expression library construction, subjected to size selection by SPRIselect reagent beads, and quality controlled for library construction via Agilent Bioanalyzer before Illumina sequencing on a HiSeq 2000.

### Long-read single-cell transcriptome sequencing

10x cDNA from the ASC and MBC gates were reamplified using forward (10x_cDNA_fwd, read1) and reverse primers (10x_cDNA_rev, TSO) (Supplemental Table S5). Additional details are provided in Supplemental Methods S3. The ASCs were prepared for sequencing following the standard Ligation Sequencing Kit (Oxford Nanopore Technologies SQK-LSK110) with short fragment buffer using 200 ng input of amplified cDNA on a PromethION (Oxford Nanopore Technologies) with R9.4.1 flow cells. The MBC gate library was prepared for sequencing following the single-cell cDNA protocol (Oxford Nanopore Technologies SQK-PCS111) using 10 ng input of enriched cDNA. The resulting single cell, whole transcriptome libraries were sequenced on a PromethION (Oxford Nanopore Technologies) with R9.4.1 flow cells and basecalled using Guppy v6.4.6 with the Kit 10 R9.4.1 super accuracy models.

### IG transcript–enriched transcriptome sequencing

10x cDNA was reamplified as described above. To increase IG heavy and light chain transcript coverage, an xGen Custom Hybridization Panel (Integrated DNA Technologies) was designed targeting IG and TCR exons. The hybridization panel consisted of 1877 5′ biotinylated probes, pooled at equimolar concentrations (Supplemental Table S6). The sample from the MBC gate was prepared starting with 500 ng of amplified 10x cDNA library. The manufacturer protocol was followed with the following modifications: no blocking oligos were used and Dynabeads kilobaseBINDER M-280 Streptavidin beads were used instead of Dynabeads M270 Streptavidin beads. Postcapture PCR details are provided in Supplemental Methods S4. After postcapture PCR, only 300 ng of the cDNA sequencing library was prepared following the Ligation Sequencing Kit (Oxford Nanopore Technologies SQK-LSK110) with short fragment buffer. The resulting single cell, IG transcript-enriched libraries were sequenced on a PromethION (Oxford Nanopore Technologies) with R9.4.1 flow cells.

### Expression analysis

Single-cell gene expression count matrices were generated from Illumina reads using Cellranger count and from Oxford Nanopore reads using the wf-single-cell pipeline (https://github.com/epi2me-labs/wf-single-cell). Data were processed in Seurat v5 (Hao et al. 2024) with platform-specific quality control thresholds for minimum cells, features, and RNA counts. All matrices were scaled, normalized to 10,000 counts per cell, and log-transformed. Additional details are provided in Supplemental Methods S5.

### Doublet analysis

Doublets were identified by *scDblFinder* (Germain et al. 2021) with default settings, using Cellranger's filtered_feature_bc_matrix for the Illumina data or wf-single-cell's gene_expression.counts.tsv for the Oxford Nanopore data. Because doublets are difficult to detect among low complexity cell populations (e.g., homotypic doublets), cell barcodes were considered doublets and removed from subsequent analysis if they were detected in either data set.

### Antibody consensus sequences

Strand-oriented and full-length reads were identified as part of the wf-single-cell pipeline. IG transcripts were identified using IgBLAST (Ye et al. 2013) with a personalized database. Reads were filtered for full length, nonchimeric reads based on quality criteria including *e*-value, CDR3 length, coverage requirements, and segment spacing, as detailed in Supplemental Figure S6. Filtered reads were grouped by locus and cell barcode, and downsampled to 150 reads per group. Consensus sequences were generated using medaka smolecule (https://github.com/nanoporetech/medaka), filtered, and validated against reference databases and our personalized assembly.

### Clonotype and mutational analysis

The Immcantation (Gupta et al. 2015) suite was used to call clones and do mutational analysis. Nearest neighbor distances were determined with the HH_S5F model (Yaari et al. 2013) and thresholds for distinguishing clones were determined using the “gamma-norm” curve. Clones were called with SCOPer (Nouri and Kleinstein 2020), using additional light chain information where available. Mutation profiling was done with SHazaM (Gupta et al. 2015).

IG network creation was done using “generate_network” from Dandelion (Suo et al. 2024), which draws the network based on the Levenshtein distance between amino acid VDJ sequences.

### Antibody synthesis

Monoclonal antibodies were produced in two batches: 45 full IgG1 antibodies and 50 scFv-Fc fusions. For batch 1, paired heavy and light chain sequences from ASC-gated cells were synthesized by GenScript and cloned into pcDNA3.4 vectors. For batch 2, sequences were synthesized as scFvs by Twist Biosciences and cloned into pFuse-hG1-Fc2 vectors. Both batches were expressed in Expi293 cells and quantified by bio-layer interferometry (BLI). Additional details are available in Supplemental Method S6.

### Neutralization assays

Vero-CCL81 (for MuV and VSV_RUBV) or Vero-hSLAM (for MeV-IC323) target cells which were seeded at a density of 2 × 10^4^ cells per well in flat-bottom 96-well plates (Thermo Fisher Scientific 08-772-3). The cells were incubated at 37°C/5% CO_2_ overnight (∼20 h). On the following day, GFP-expressing vaccine strain MuV-JL5, recombinant MeV-IC323, or recombinant VSV virions expressing Rubella E2/E1 envelope region were preincubated with serially diluted antibodies for a minimum of 30 min at 37°C (MuV and MeV-IC323) or for 1 h at 32°C (for VSV_RUBV) and added to the target cells. Between 16 and 24 h postinfection, GFP counts were acquired by the Celigo imaging cytometer (Nexcelom Biosciences, version 4.1.3.0)

### ELISA

ELISA kits against MMR were purchased commercially (Arlington Scientific 850096AG, 860096AG, and 800096AG) and performed according to the manufacturer's instructions. Antigen substrates were sonicated infected cell lysates. Briefly, the antibody samples and the various controls were first diluted, then added to the wells. The plates were then incubated at room temperature for 30 min, washed 4×, after which HRP-conjugated secondary antihuman antibodies were added and the plates incubated for another 30 min at room temperature. Wells were then washed four more times; HRP substrate added and incubated an additional 30 min at room temperature. Stop reagent was added and absorbance was read by the CYTATION Gen5 Microplate reader (BioTek, part of Agilent Technologies, version 1.15) at 405 nm against reagent blank.

### Cell surface binding assays

Viral envelope protein binding was evaluated using three methods. For initial screening, Expi293 cells were transfected with pCAGG expression plasmids encoding mumps and measles envelope proteins. After 48 h, cells were washed, resuspended, and incubated with recombinant antibodies, followed by detection with fluorescent antihuman IgG antibodies. For validation of MeV-binding antibodies, Raji-DC-SIGN cells were infected with measles virus (MOI 0.01) for 3–5 days, then incubated with antibodies at varying dilutions. Binding analysis was done via flow cytometry. FCS files were analyzed with FlowJo v10.

HCMV binding was assessed using human dermal fibroblasts infected with HCMV-GFP (AD169 strain, MOI 0.2). After 72 h, cells were fixed, permeabilized, and stained with MMR antibody clones (1 μg). Binding was detected using fluorescently labeled secondary antibodies and quantified by imaging cytometry, with Cytogam serving as a positive control. Additional details are provided in Supplemental Method S7.

#### AI statement

Portions of this manuscript were reworded for clarity with assistance from Claude 3.5 Sonnet (Anthropic, 2024). All content was manually reviewed for accuracy.

## Data access

The sequencing data generated in this study have been submitted to the NCBI BioProject database (https://www.ncbi.nlm.nih.gov/bioproject/) under accession number PRJNA1087553. A pipeline for consensus generation, assembly, annotation, and clonotyping is available at GitHub (https://github.com/LynnLy/ig_consensus_pipeline) and as Supplemental Code.

## Supplemental Material

Supplement 1

Supplement 2

Supplement 3

Supplement 4
