# Supplementary material for: De novo antibody identification in human blood from full-length single B cell transcriptomics and matching haplotype-resolved germline assemblies
Source: Genome Res. 2025 Apr;35(4):929–41. doi: 10.1101/gr.279392.124 (PMC12047243; doi:10.1101/gr.279392.124)
Supplement: Supplement 4 [file Supplemental_Material.pdf]

Supplementary Materials

Table of Contents

Figure S1: Novel *IGLC* Allele 2
Figure S2: *IGH* Methylation 3
Figure S3: FACS plots 4
Figure S4: ASC gate UMI counts per cell barcode 5
Figure S5: MBC gate UMI counts per cell barcode 6
Figure S6: Glycosylation gene expression 7
Figure S7: IG consensus pipeline 8
Figure S8: Isotype lengths 9
Figure S9: *IGH* consensus accuracy in CH1 region 10
Figure S10: ELISA binding and neutralization assay results 11
Figure S11: Cell Surface Binding assay results 12
Figure S12: HCMV binding assay results 13
Figure S13: V-gene usage in light chains 14
Figure S14: J-gene usage 15
Figure S15: J-gene usage for IgD-Only MBC 16
Figure S16: Ambiguous V gene allele assignment 17
Table S1: Whole genome assembly stats 18
Table S2: Novel germline allele usage 19
Table S3: Differentially expressed transcripts in MBC gate 20
Table S4: Nanopore sequencing statistics 21
Table S5: Primers for amplification of 10x libraries 22
Table S6: Probe sequences 23
Methods S1: FACS Cocktail Contents 24
Methods S2: Germline Immune Repertoire Annotation 24
Methods S3: Long Read Single-cell Transcriptome Sequencing 24
Methods S4: IG Transcript-enriched Transcriptome Sequencing 25
Methods S5: Expression Analysis 25
Methods S6: Antibody Synthesis 25
Methods S7: Cell Surface Binding Assays 26

Figure S1: Novel *IGLC* Allele

IGV pileups of closest matching reference alleles for four novel alleles present in the IG assembly. The colored vertical bars are mismatches between the assembled contig and the reads or alleles. Upper: Raw reads aligned to the assembly. The reads support the assembled sequence, without heterogeneity or decreased QV scores at the novel SNP sites. Lower: Known reference alleles aligned to the assembly, sorted by alignment score. The closest known matches have at least 1 SNP difference from the assembly and the genomic reads.

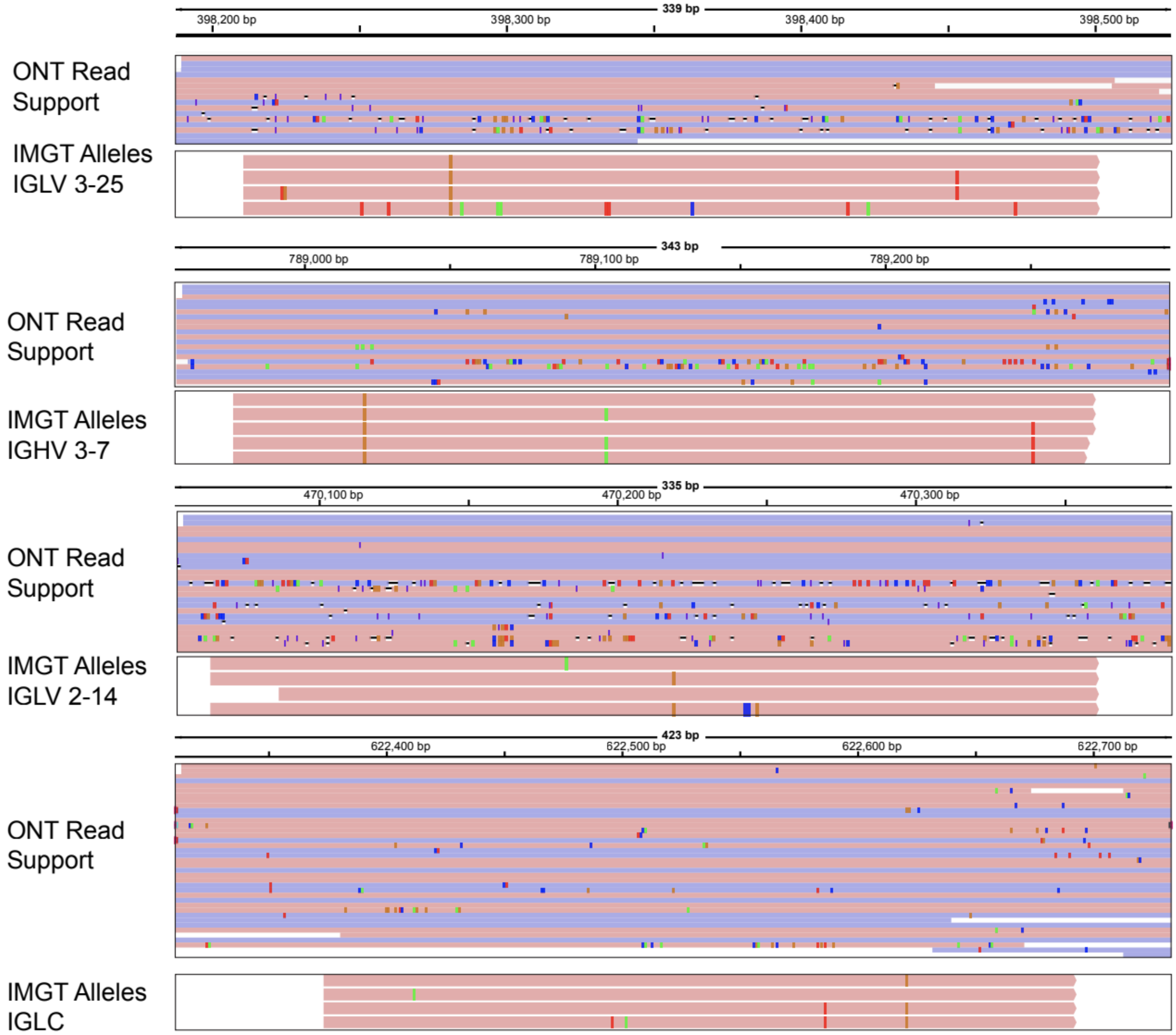

Figure S2: *IGH* Methylation

Methylation frequencies across all *IGHV* genes and around the *IGHD* and *IGHJ* loci. For *IGHV*, methylation was aggregated across CpGs based on their distance from the nearest *IGHV* transcription start site (TSS). The curves depicted were generated using LOESS smoothing (span = 0.1).

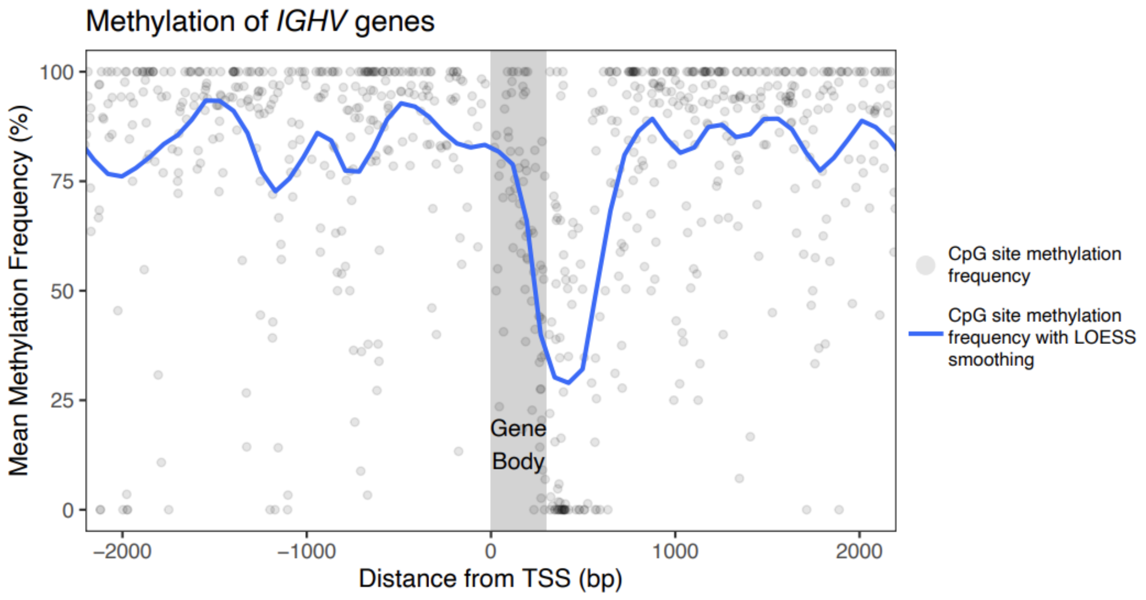

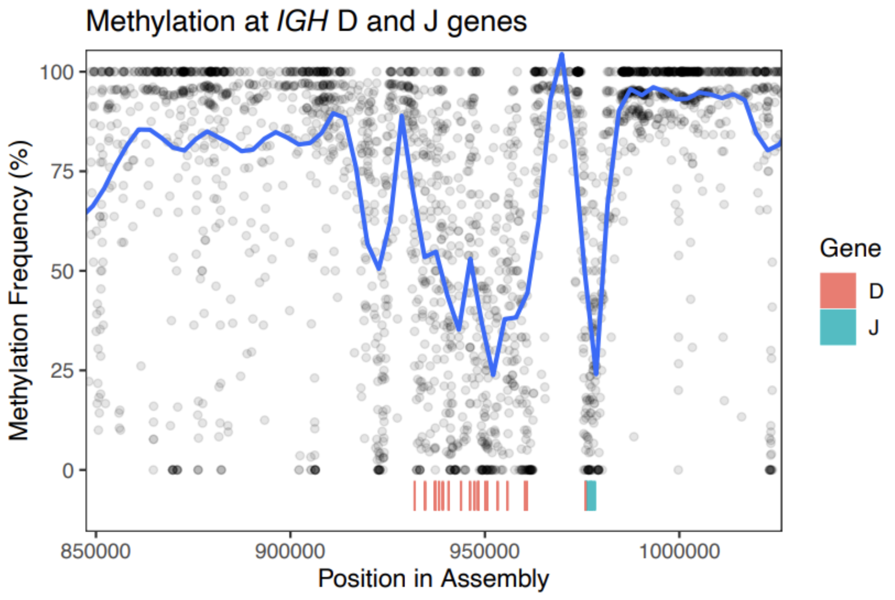

Figure S3: FACS plots

PBMCs were first enriched for B cells via negative selection and sorted for memory B cells and antibody-secreting B cells according to the strategy shown. B cell enriched PBMCs were gated to retain only singlets and live cells (P4 gate). Cells in the P4 gate were then gated on $CD19^+$ B cells that were IgM^-. A cocktail of antibodies corresponding to T cells (CD3/CD4/CD8) and monocytes/NK cells (CD16) were further added to ensure purity of $CD19^+/IgM^-$ B cells (P5 gate). P5 gate cells were sorted for antibody-secreting cells ($CD38^{++}/CD27^{++}$) and memory B cells ($CD38^-/CD27^+$) in the P7 gate (referred to as ASC gate) and P8 gate (referred to as MBC gate), respectively.

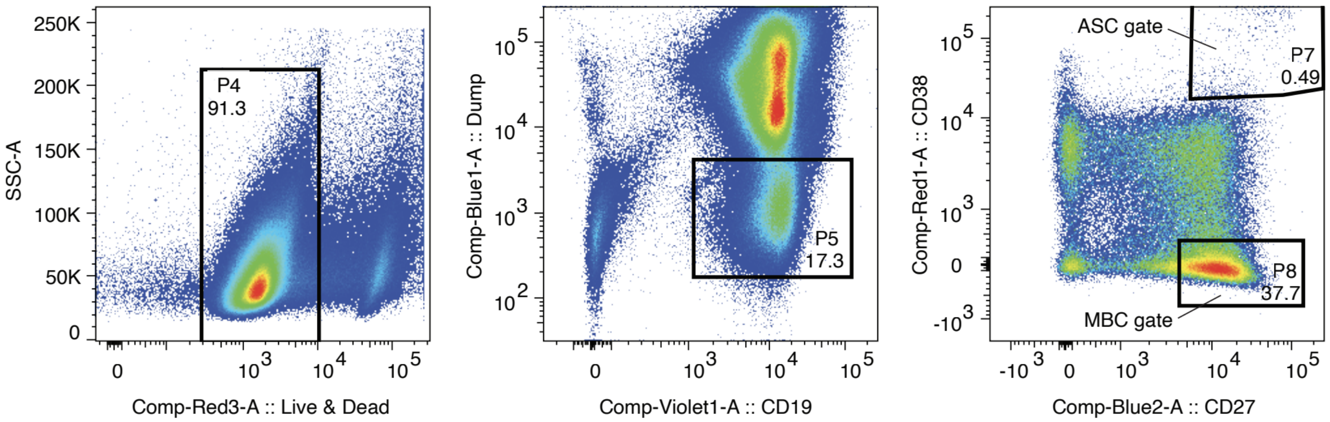

Figure S4: ASC gate UMI counts per cell barcode

UMI counts per cell barcode in Illumina and ONT for the single-cell library generated from the FACS P7 (ASC) gate.

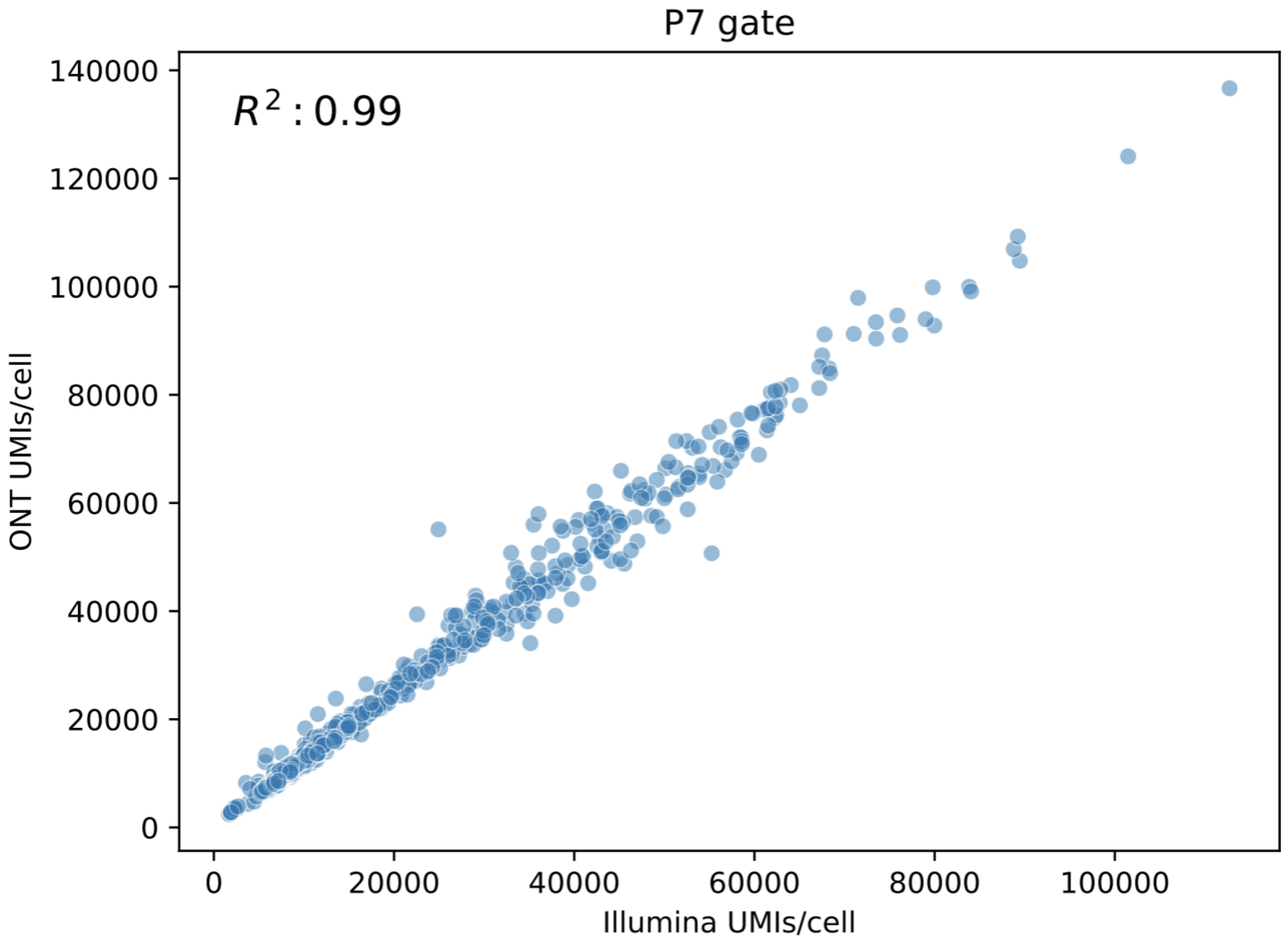

Figure S5: MBC gate UMI counts per cell barcode

UMI counts per cell barcode in Illumina and ONT for the single-cell library generated from the FACS P8 (MBC) gate.

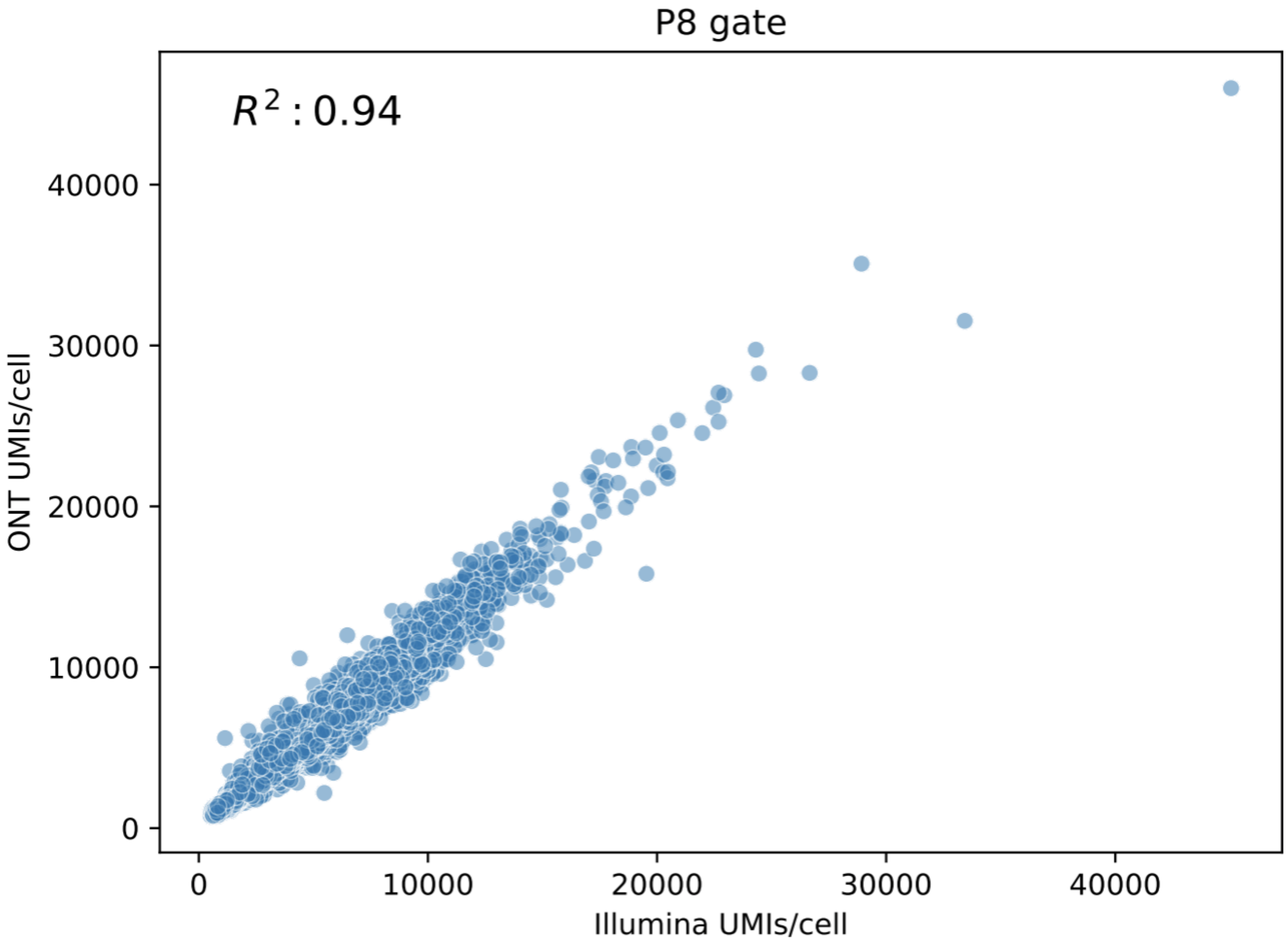

Figure S6: Glycosylation gene expression

Expression of glycosylation genes in memory B cells from the FACS MBC gate.

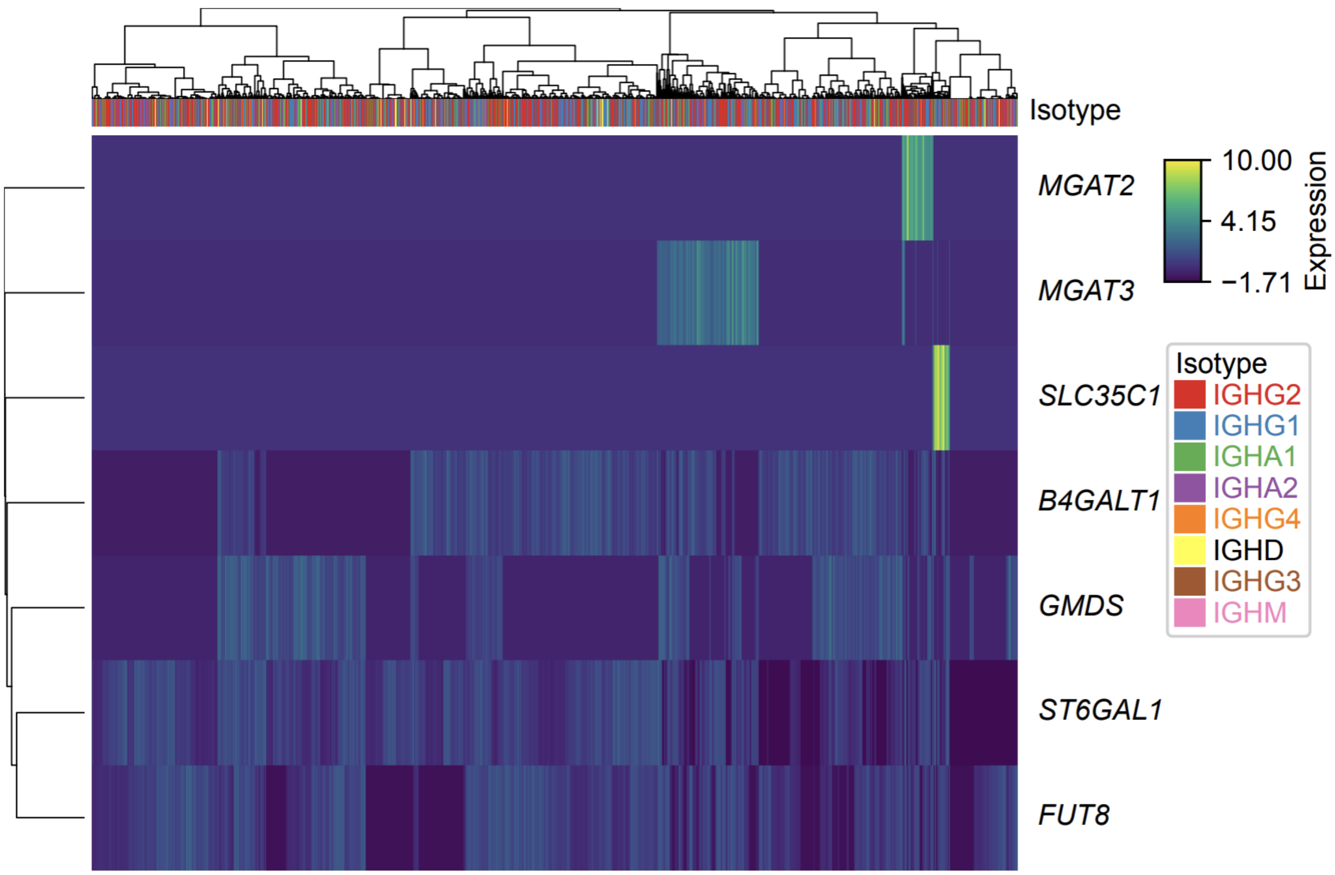

Figure S7: IG consensus pipeline

Pipeline used to create consensus heavy and light chain sequences for each cell barcode. Reads were queried against a personalized database using IgBLAST (Ye et al. 2013) with parameters "-c_region_db ncbi_human_c_genes -auxiliary_data human_gl.aux -outfmt 19". Quality filtering criteria:

1. V and C segment e-values <1e-6
2. CDR3 length 3-50 amino acids
3. V and C segment coverage >90%
4. V-C segment separation <250 bp
5. <400 bp flanking sequence before V or after C segments

Filtered reads were grouped by IGK, IGL, or IGH locus and cell barcode, then downsampled to maximum 150 reads per group. Consensus sequences were generated using medaka (github.com/nanoporetech/medaka) with parameters "--min_depth 3 --depth 10 --model r941_e81_sup_g514". Final validation used IgBLAST queries against IMGT GENE-DB (Giudicelli et al. 2005), OGRDB (Lees et al. 2020), and our personalized assembly-based database. For cells with multiple light chains, only the highest coverage sequence was retained. Sequences were filtered if unproductive, incomplete, or missing conserved junction anchors (C104 and W/F116).

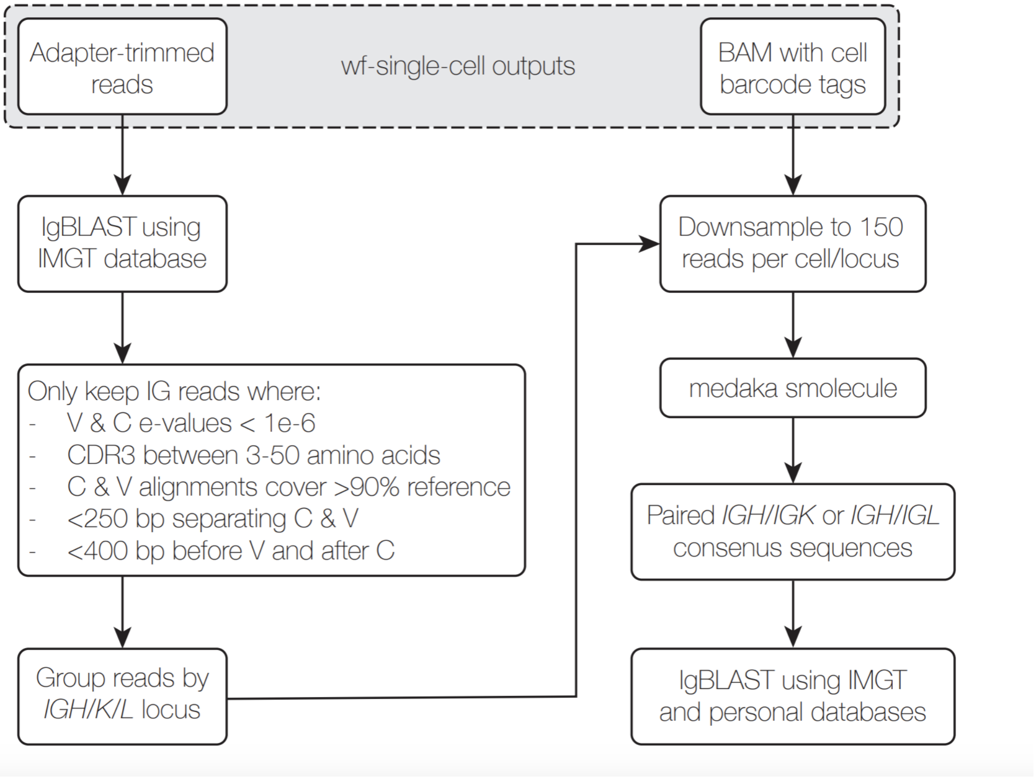

Figure S8: Isotype lengths

Nucleotide consensus coding-sequence length distributions by locus (*IGH*, *IGK*, and *IGL)* and isotype.

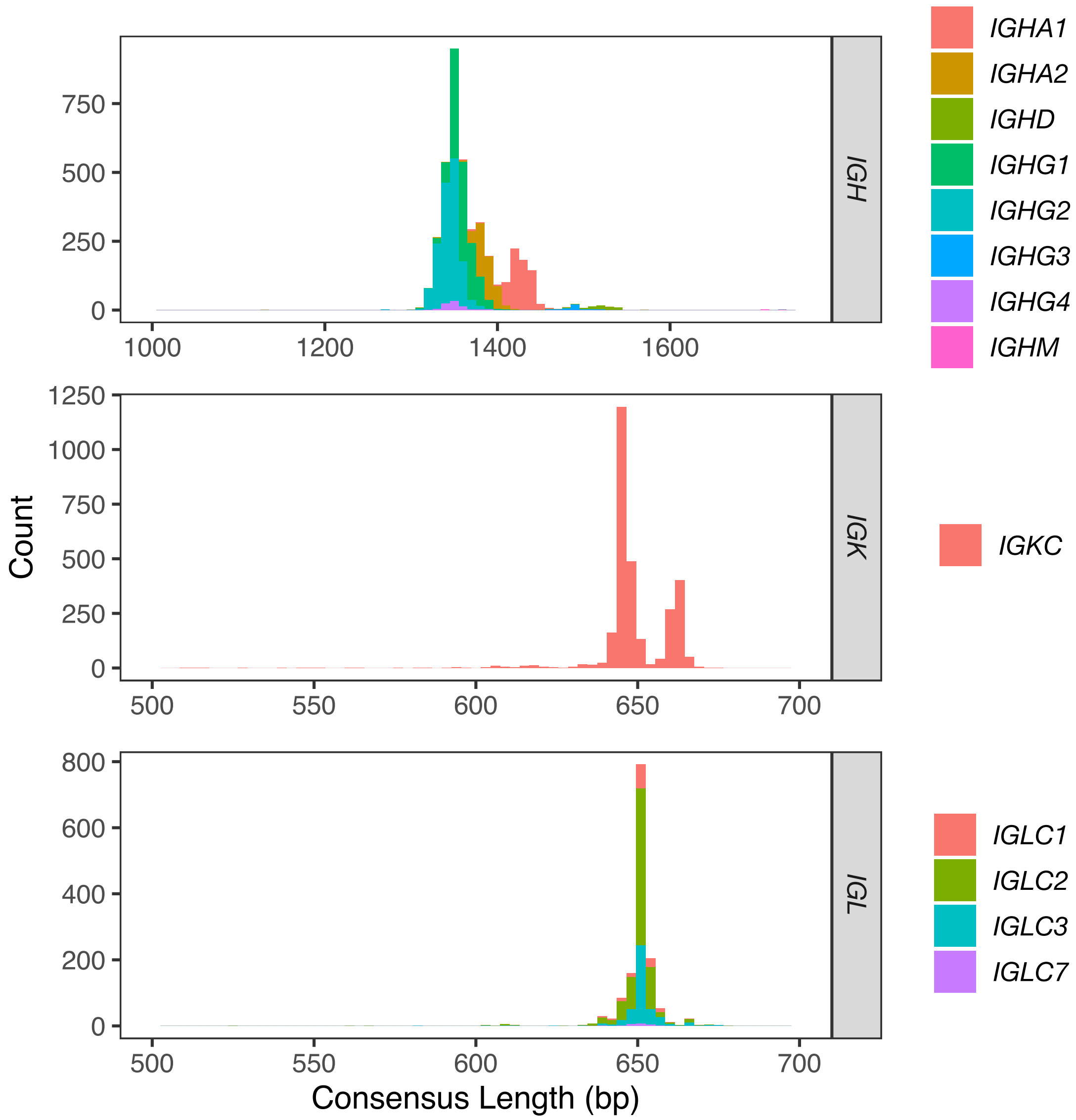

Figure S9: *IGH* consensus accuracy in CH1 region

Error rate of *IGH* consensus sequences based on the conserved CH1 region. CH1 alleles present in the germline assembly were aligned to passing consensus sequences. 3883 out of 4040 consensus sequences (96%) have 0 errors in the CH1 region.

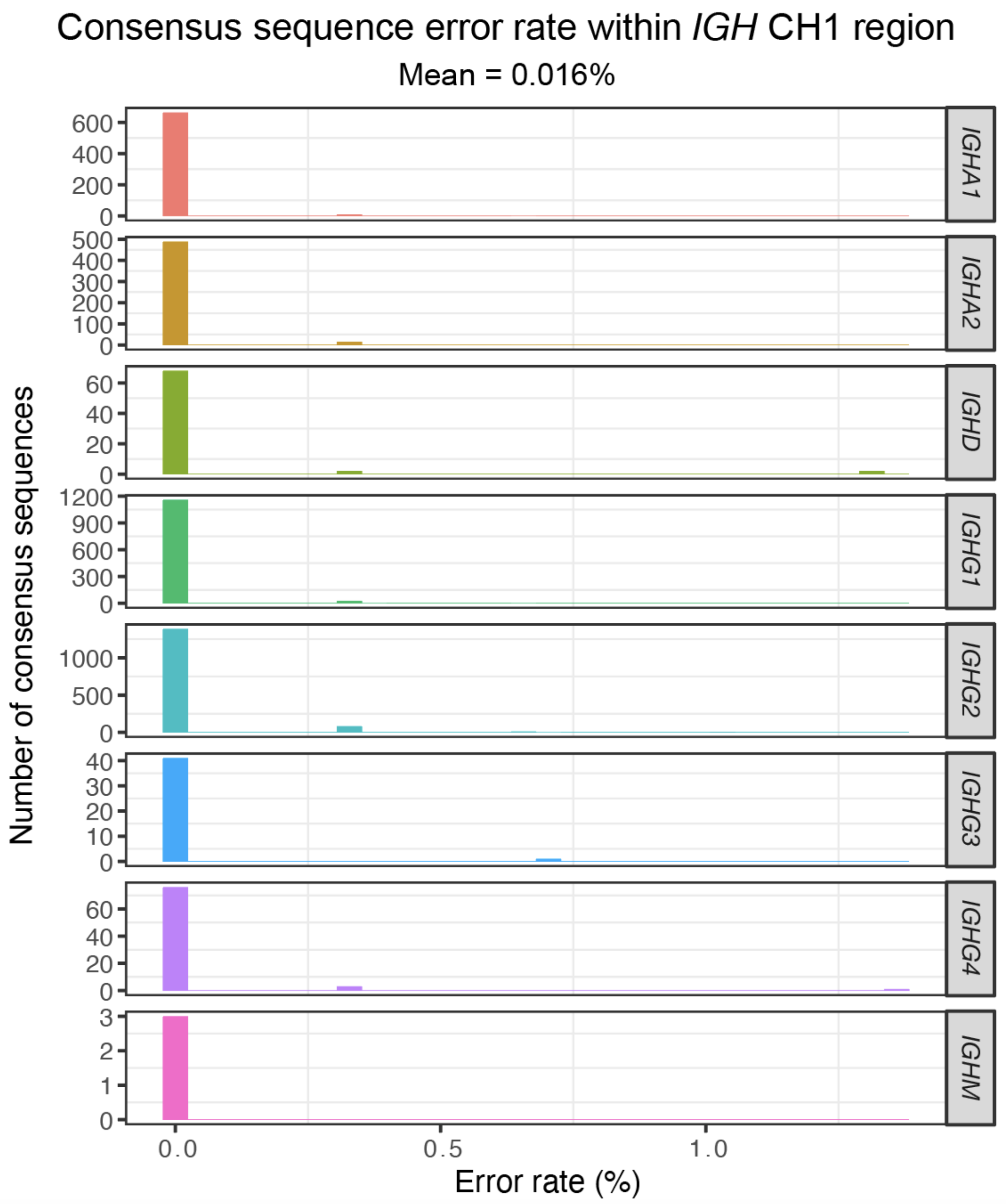

Figure S10: ELISA binding and neutralization assay results

ELISA binding results for Measles (A), Mumps (B) and Rubella (C) virus lysate performed as described in methods. Corresponding neutralization assay results for Measles (D), Mumps (E) and Rubella (F) virus entry performed as described in methods. For Rubella virus, only a subset of antibodies were tested for neutralization although all antibodies were tested for binding in (C).

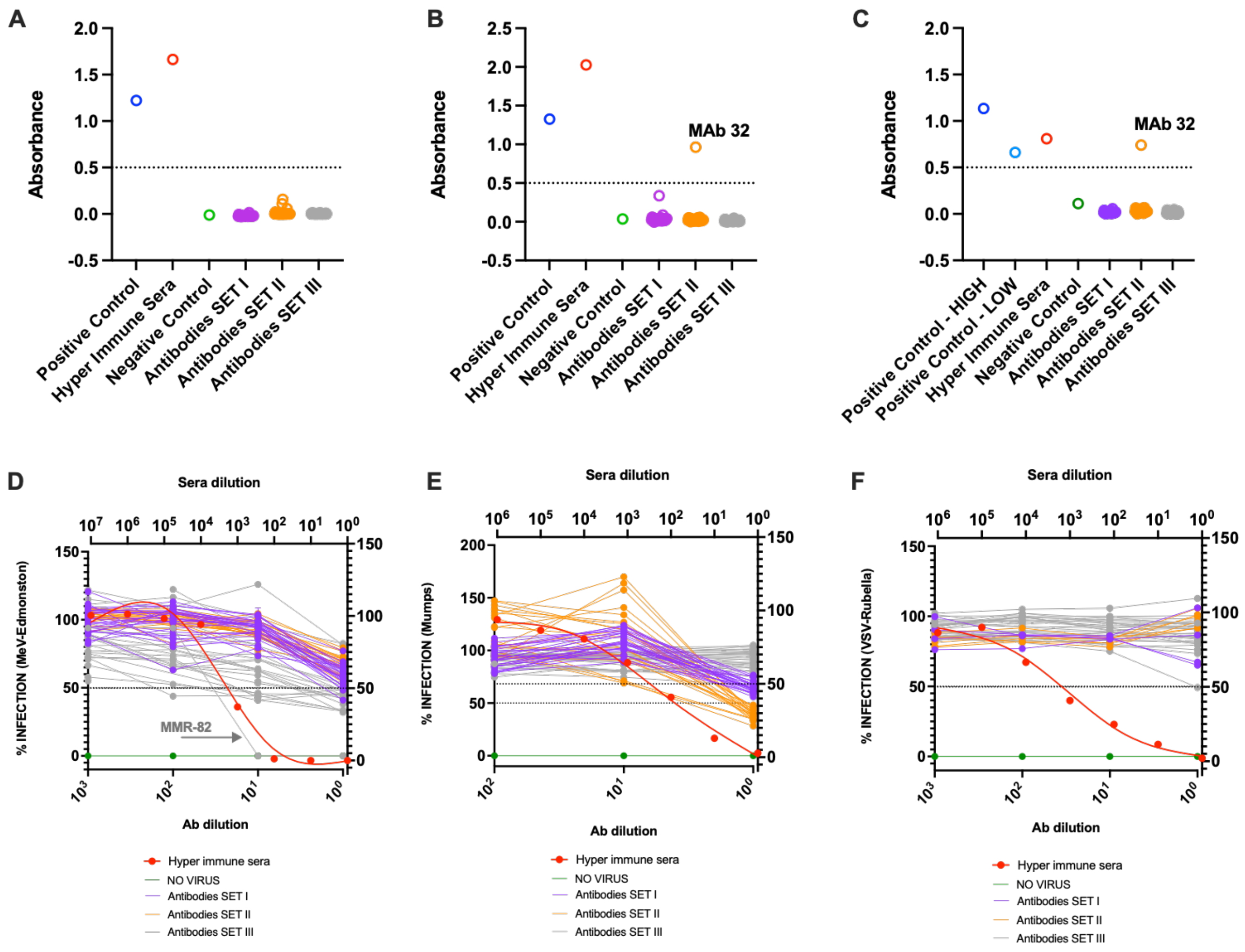

Figure S11: Cell Surface Binding assay results

ExpiCHO cells were transfected with pCAGG mammalian expression constructs containing MeV F, MeV RBP, MuV F or MuV HN proteins and stained with the ScFv-human G1-Fc monoclonal antibodies from our second batch of clones. Stained cells were detected with anti-human IgG-APC secondary and mean fluorescent intensities were recorded. Graph represents a select number of the clones, including the positive binders. Mock (empty plasmid) transfected cells and staining with no primary antibody (No Ab) were used as negative controls.

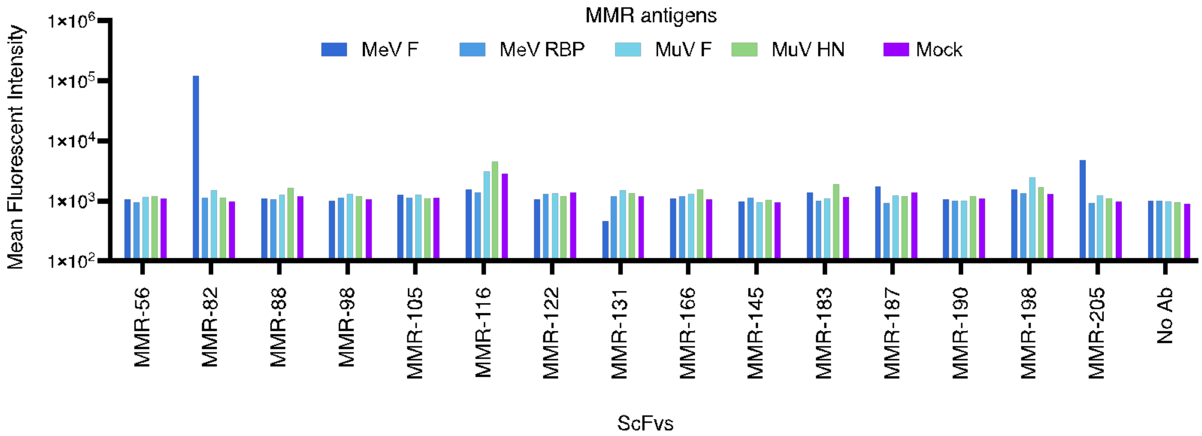

Figure S12: HCMV binding assay results

A HCMV-GFP reporter virus (AD169) was used to infect normal human dermal fibroblasts. The binding of the primary MMR mAb was detected by the secondary AlexaFluor-647 goat-Anti human IgG [H+L]. Cytogam (pooled CMV+ IgG) was used as a positive control.

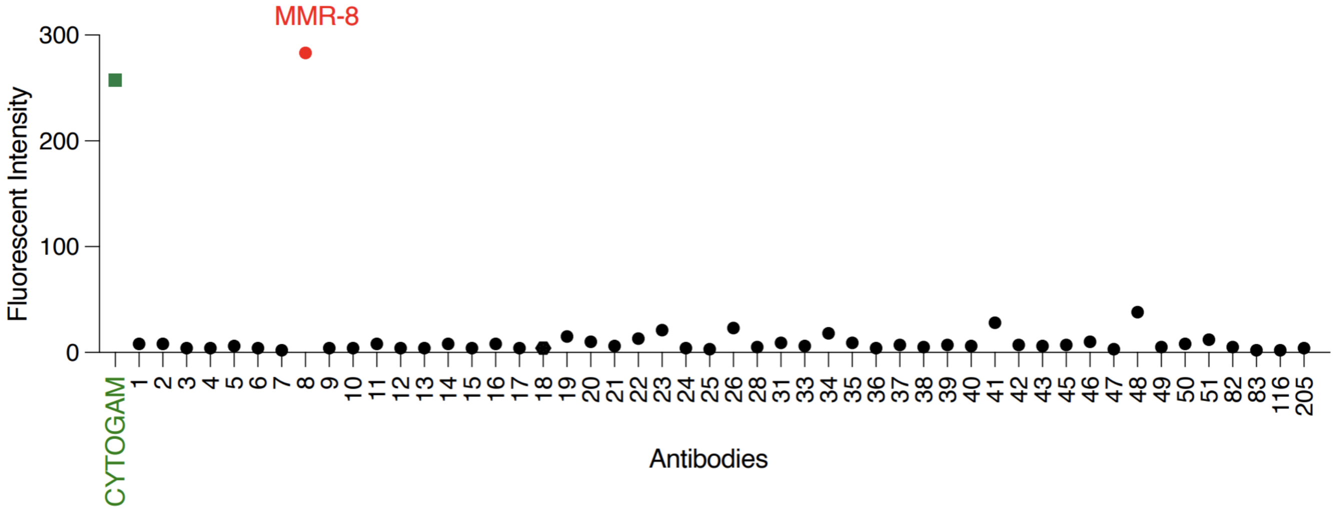

Figure S13: V-gene usage in light chains

V-gene distribution in light chains. V-genes were assigned by IgBLAST using the personalized database for *IGL* and the default IMGT database for *IGK*. As for Figure 6A, sequences that blasted with less than 85% VK or VL identity (light blue) were analyzed separately.

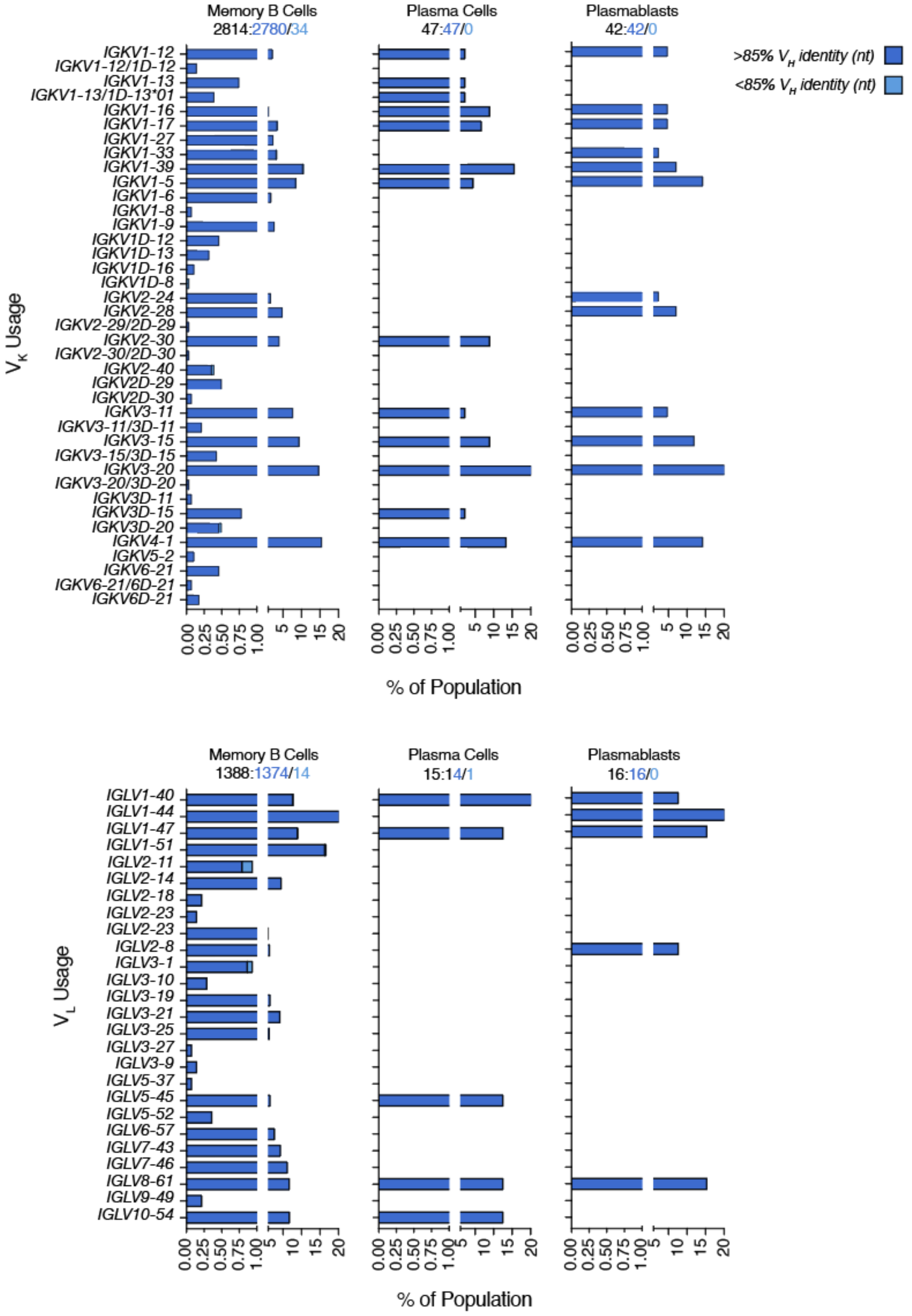

Figure S14: J-gene usage

J-gene distribution in light and heavy chains. MG: Ambiguous call between multiple genes.

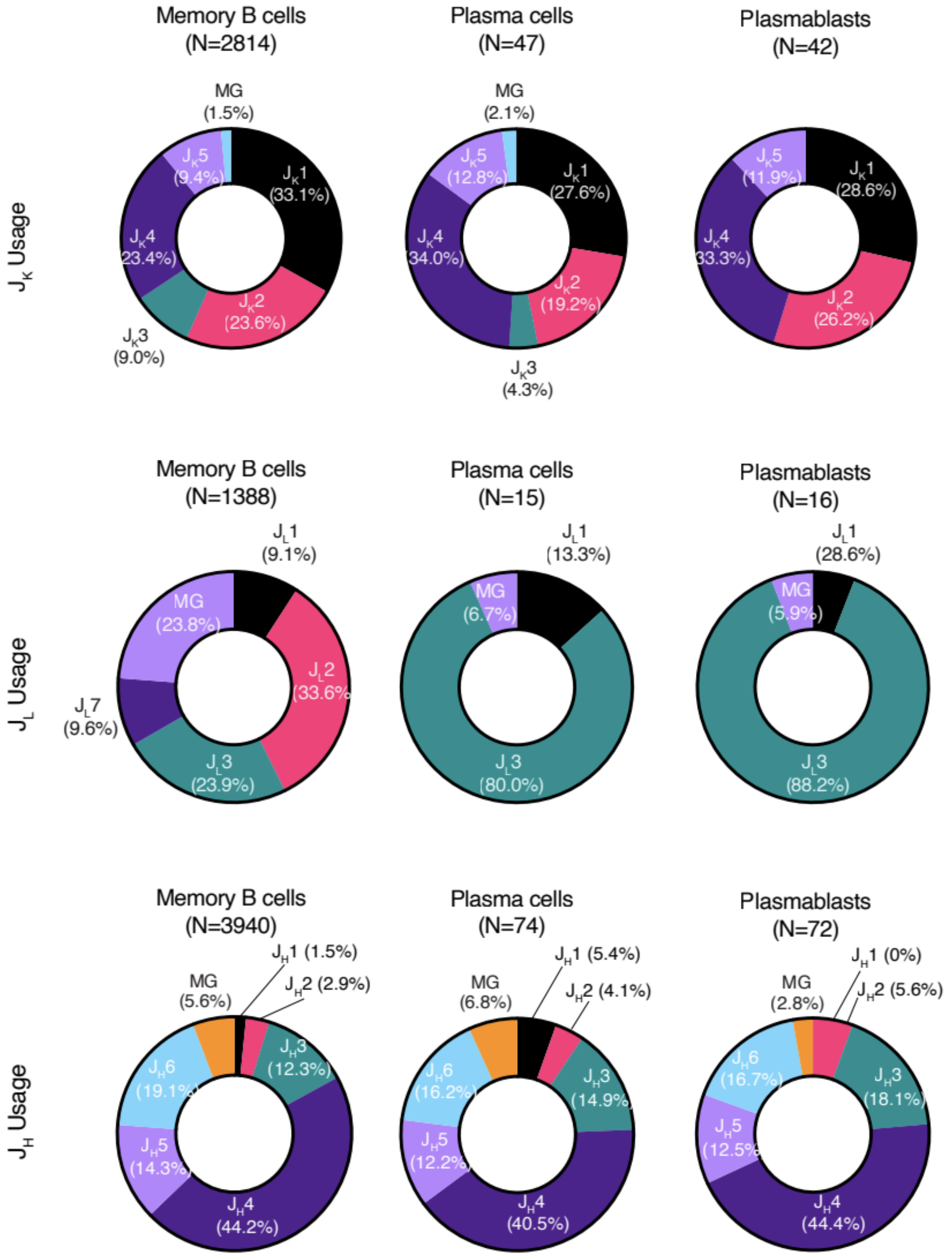

Figure S15: J-gene usage for IgD-Only MBC

J-gene distribution for IgD-only MBC heavy chains compared to IgG1 MBCs. MG: Ambiguous call between multiple genes.

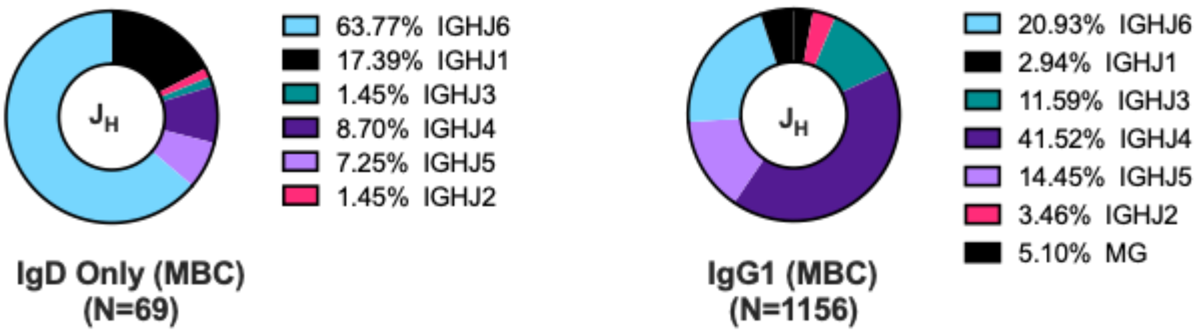

Figure S16: Ambiguous V gene allele assignment

Reference alleles were first deduplicated by sequence. When using the IMGT-GENE database with IgBLAST, 14% of all functional V allele calls representing 57 different *IGH* and *IGL* genes are ambiguously assigned to multiple equally likely alleles. 90% of these ambiguous V allele calls have a clear best-match when using the personalized database.

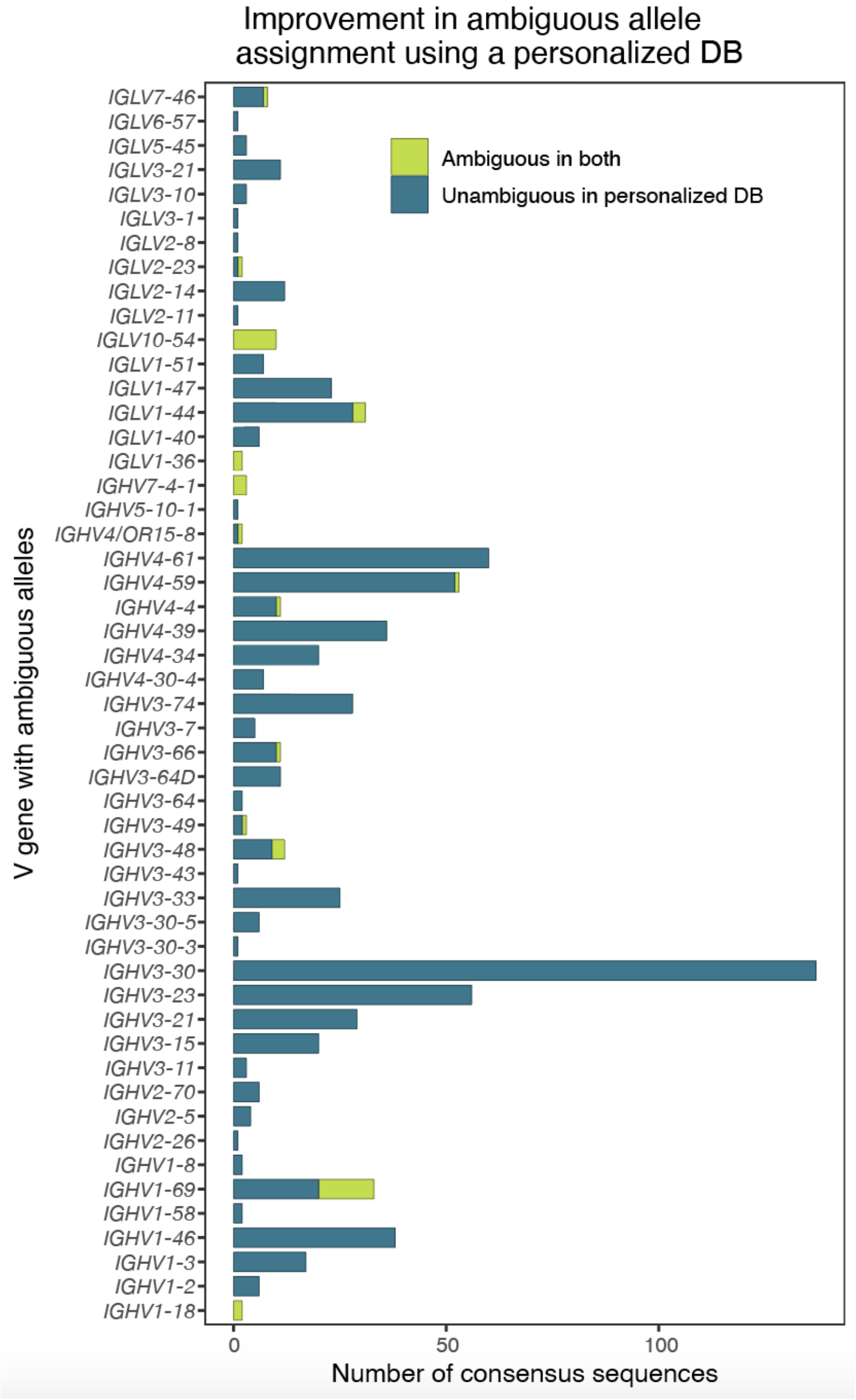

Table S1: Whole genome assembly stats

Whole genome assembly statistics from Flye and Margin (as a part of the HapDup pipeline).

Total Length	Fragments	Fragments N50	Largest Fragment	Phase Sets	Phase Set N50	Largest Phase Set	Average Phase Set	Variants	Phased Variants
2.87 Gb	2,437	28.5 Mb	109 Mb	9,791	1.0 Mb	5.4 Mb	252 Kb	3,218,774	2,766,421

Table S2: Novel germline allele usage

Novel germline allele type and usage in the consensus sequences. Novel alleles with substitutions were used in ~1% of filtered consensus sequences.

Locus	Closest existing allele	Type	Amino Acid Mutation	Effect on Ag binding	# Cells Expressing	# Transcripts
IGH V	IGHV3-7*03	substitution	G -> R in FR1 (aa17)	Likely no effect	70	4736
IGL V	IGLV2-14*03	substitution	E -> D in CDR2 (aa56)	Might affect Ag binding	11	174789
IGL V	IGLV3-25*03	substitution	G -> A in FR1 (aa25)	Likely no effect	1	20186
IGL C	IGLC3*04	substitution			*	

*One novel allele differed from IGLC*04 by 1 substitution, but IgBLAST only categorizes C genes to gene resolution and not allele resolution.

Table S3: Differentially expressed transcripts in MBC gate

Transcripts whose expression in one cluster was significantly different from the expression in all cells outside of that cluster. Calculated using the FindAllMarkers function in Seurat. Only showing a sample of the table; full table available in Supplemental_Table_S3.xlsx.

*p*_val	avg_log2FC	pct.1	pct.2	*p*_val_adj	cluster	transcript	gene
0	1.713124038	0.873	0.425	1.00E-300	0	ENST00000390237	*IGKC*
0	-3.827342595	0.238	0.712	1.00E-300	1	ENST00000390237	*IGKC*
0	1.45942445	0.805	0.325	1.00E-300	0	ENST00000390239	*IGKJ4*
0	-4.644802866	0.126	0.632	1.00E-300	1	ENST00000390239	*IGKJ4*
0	5.221164075	0.926	0.046	1.00E-300	4	ENST00000390243	*IGKV4-1*
0	7.607260953	0.435	0.003	1.00E-300	6	ENST00000390282	*IGLV4-69*
0	9.72600845	0.831	0.002	1.00E-300	11	ENST00000390287	*IGLV10-54*
0	6.256240211	0.278	0.006	1.00E-300	5	ENST00000390295	*IGLV7-46*
0	6.348209057	0.774	0.062	1.00E-300	5	ENST00000390298	*IGLV7-43*
0	8.888787092	0.928	0.015	1.00E-300	7	ENST00000390310	*IGLV2-18*
0	6.788620741	0.404	0.006	1.00E-300	7	ENST00000390314	*IGLV2-11*
0	5.785336229	1	0.089	1.00E-300	7	ENST00000390321	*IGLC1*
0	-4.115259177	0.16	0.622	1.00E-300	0	ENST00000390323	*IGLC2*
0	2.777203054	0.9	0.289	1.00E-300	1	ENST00000390323	*IGLC2*
0	-3.956644037	0.167	0.589	1.00E-300	0	ENST00000390325	*IGLC3*
0	2.560879114	0.848	0.282	1.00E-300	1	ENST00000390325	*IGLC3*
0	6.985236444	0.935	0.037	1.00E-300	6	ENST00000390331	*IGLC7*
0	7.620384726	0.96	0.017	1.00E-300	10	ENST00000390556	*IGHD*
0	8.400548374	0.932	0.004	1.00E-300	8	ENST00000448155	*IGKV1D-39*
0	8.638278818	0.778	0.003	1.00E-300	9	ENST00000468494	*IGKV2-30*
0	8.99866742	0.713	0.001	1.00E-300	9	ENST00000474213	*IGKV2D-30*
0	1.81024006	0.84	0.307	1.00E-300	2	ENST00000507116	*PDE4D*

Table S4: Nanopore sequencing statistics

Nanopore sequencing statistics for the single-cell libraries and the germline genome library.

Library	Sample	Library treatment	Kit	# Flow cells	Flow cell ID	Base calling mode	Total reads (M)	Total yield (Gb)	Read N50 (bp)
Single-cell RNA sequencing	P7 gate: Antibody secreting cells	Whole transcriptome	LSK110	2	PAI05989	Simplex	70.4	84.3	1,336
					PAI07242	Simplex	99.9	111.9	1,195
	P8 gate: Memory B cells	Whole transcriptome	PCS111	2	PAM80546	Simplex	182.1	140.7	830
					PAM71314	Simplex	187.8	144.1	825
		IG-enrichment	LSK110	1	PAI92332	Simplex	123.7	129.3	1,054
Germline genome sequencing	Monocytes	Whole genome	LSK114	1	PAQ76345	Simplex	16.9	125.4	21,173
						Duplex	1.6	12.8	20,701

Table S5: Primers for amplification of 10x libraries

Primer sequences used to amplify the 10x single-cell cDNA library prior to sequencing.

Primer name	Primer Sequence
10x_cDNA_fwd	CTACACGACGCTCTTCCGATCT
10x_cDNA_rev	AAGCAGTGGTATCAACGCAGAG

Table S6: Probe sequences

Probe sequences from the xGen™ Custom Hybridization Panel (Integrated DNA Technologies) targeting BCR and TCR exons. The panel consisted of 1877 5' biotinylated xGen Lockdown Probes, pooled at equimolar concentrations and purified with standard desalting. Only showing a sample of the table; full table available in Supplemental_Table_S6.xlsx.

Sequence Name	Sequence
783320_45035616_IGKV1OR-2|exon_2|ENSG00000156755_1_1	/5Biosg/GTG CCA GAT GTG ACA TCC AGA TGA CCC AGT CTC CAT CCT CCC TGT CTG CAT CTG TAG GAG GCA GAG TCA CCA TCA CTT GCC GGG CGA GTC AGG GCA TTA GCA ATA ATT TAA ATT GGT ATC
783320_45035616_IGKV1OR-2|exon_2|ENSG00000156755_1_2	/5Biosg/CAG GGC ATT AGC AAT AAT TTA AAT TGG TAT CAG CAG AAA CCA AGG AAA ACT CCT AAG CTC CTG ATC TAT GCT GCA TCC AGT CTG CAA AGT GGG ATT CCC TCT CGG TTC AGT GAC AGT GGA
783320_45035616_IGKV1OR-2|exon_2|ENSG00000156755_1_3	/5Biosg/TGG GAT TCC CTC TCG GTT CAG TGA CAG TGG ATC TGG GAC AGA TTA CAC TCT CAC CAT CAG CAG CCT GCA GCC TGA AGA TTT TGC AAC TTA TTA CTG TCA ACA GAG TGA CAG TAA CCC TCC
783320_45035617_TRBV21OR9-2|exon_1|ENSG00000183938_1_1	/5Biosg/CCT CCA TGG ACA CCA AGG TCA CCC AGA GAC CTA GAT TTC TGG TCA AAG CAA ATG AAC AGA AAG CAA AGA TGG ACT GTG TTC CTA TAA AAA GAC ATA GTT ATG TTT ACT GGT ATC ATA AGA
783320_45035617_TRBV21OR9-2|exon_1|ENSG00000183938_1_2	/5Biosg/AAG ACA TAG TTA TGT TTA CTG GTA TCA TAA GAC GCT GGA AGA AGA GCT CAA GTT TTT TAT TTA CTT TCA GAA TGA AGA AAT TAT TCA GAA AGC AGA AAT AAT CAA TGA GCG ATT TTC AGC
783320_45035617_TRBV21OR9-2|exon_1|ENSG00000183938_1_3	/5Biosg/AAA GCA GAA ATA ATC AAT GAG CGA TTT TCA GCC CAA TGC CCC CAA AAC TCA CCC TGT ACC TTG GAG ATC CAG TCC ACG GAG TCA GGA GAC ACA GCA CGG TAT TTC TGT GCC AAC AGC AAA
783320_45035618_IGHV1OR15-9|exon_2|ENSG00000188403_1_1	/5Biosg/GTG CCC AGT CCC AGG TAC AGC TGA TGC AGT CTG GGG CTG AGG TGA AGA AGC CTG GGG CCT CAG TGA GGA TCT CCT GCA AGG CTT CTG GAT ACA CCT TCA CCA GCT ACT GTA TGC ACT GGG
783320_45035618_IGHV1OR15-9|exon_2|ENSG00000188403_1_2	/5Biosg/CTT CAC CAG CTA CTG TAT GCA CTG GGT GTG CCA GGC CCA TGC ACA AGG GCT TGA GTG GAT GGG ATT GGT GTG CCC TAG TGA TGG CAG CAC AAG CTA TGC ACA GAA GTT CCA GGG CAG AGT
783320_45035618_IGHV1OR15-9|exon_2|ENSG00000188403_1_3	/5Biosg/TAT GCA CAG AAG TTC CAG GGC AGA GTC ACC ATA ACC AGG GAC ACA TCC ATG GGC ACA GCC TAC ATG GAG CTA AGC AGC CTG AGA TCT GAG GAC ACG GCC ATG TAT TAC TGT GTG AGA GA
783320_45035619_IGKV7-3|exon_1|ENSG00000197794_1_1	/5Biosg/GGG ACA TTG TGC TGA CCC AGT CTC CAG CCT CCT TGG CCG TGT CTC CAG GAC AGA GGG CCA CCA TCA CCT GCA GAG CCA GTG AGA GTG TCA GTT TCT TGG GAA TAA ACT TAA TTC ACT GGT
783320_45035619_IGKV7-3|exon_1|ENSG00000197794_1_2	/5Biosg/AGT TTC TTG GGA ATA AAC TTA ATT CAC TGG TAT CAG CAG AAA CCA GGA CAA CCT CCT AAA CTC CTG ATT TAC CAA GCA TCC AAT AAA GAC ACT GGG GTC CCA GCC AGG TTC AGC GGC AGT
783320_45035619_IGKV7-3|exon_1|ENSG00000197794_1_3	/5Biosg/CAC TGG GGT CCC AGC CAG GTT CAG CGG CAG TGG GTC TGG GAC CGA TTT CAC CCT CAC AAT TAA TCC TGT GGA AGC TAA TGA TAC TGC AAA TTA TTA CTG TCT GCA GAG TAA GAA TTT TCC
783320_45035620_IGKV1OR2-3|exon_1|ENSG00000204670_1_1	/5Biosg/TGA CAT CCA GAT GAC CCA GCC TCC ATC CTC CCT GTC TGC ATC TGT AGG AGA CAG AGT CAC CGT CTC TTG CCA GGC TAG TCA AAG CAT TTA CAA CTA TTT AAA TTG GTA TCA GCA GAA ACC
783320_45035620_IGKV1OR2-3|exon_1|ENSG00000204670_1_2	/5Biosg/CAT TTA CAA CTA TTT AAA TTG GTA TCA GCA GAA ACC AGG GAA AGC ACC TAA GTT CCT GAC CTA TAG GGC ATC CAG TTT GCA GAG GGG GAT GCC ATC TCA GTT CAG TGG CAG CGG ATA TGG

Methods S1: FACS Cocktail Contents

The antibody-secreting / memory B cell specific FACS cocktail contained: Ovalbumin-AF488 (Thermo Fisher Scientific), mouse anti-human IgM-AF488 (clone SA-DA4, SouthernBiotech, Birmingham, AL), mouse anti-human CD3-FITC (clone 7D6, Thermo Fisher Scientific, mouse anti-human CD4-AF488 (clone RFT4, SouthernBiotech), mouse anti-human CD8-AF488 (clone RFT8, SouthernBiotech), mouse anti-human CD16-AF488 (clone 3G8, SouthernBiotech), mouse anti-human CD19-BV421 (clone HIB19, BD), mouse anti-human CD27-PE (clone O323, BioLegend), and mouse anti-human CD38-AF647 (clone HIT2, BioLegend).

Methods S2: Germline Immune Repertoire Annotation

A pipeline to assemble and annotate IG loci from whole genome sequence data is available at https://github.com/LynnLy/ig_consensus_pipeline. WGS reads were assembled into a haploid assembly using Flye (Kolmogorov et al. 2019) (--nano-hq). The assembly was converted into a diploid assembly using the HapDup (Kolmogorov et al. 2023) pipeline. Assembly validation and structural variant analysis was performed by mapping contigs to GRCh38 using minimap2 v2.24 (Li 2018) with `-ax asm10`. Sniffles2 v2.2 (Smolka et al. 2024) was used for structural variant calling to identify potential misassemblies. Based on the structural variant analysis, the known 5-10-1 / 3-64D alternate contig was incorporated into haplotype 2 using breakpoint coordinates that optimized raw read alignment across the locus.

For annotation of immunoglobulin gene segments, we used distinct BLASTN parameters optimized for the different lengths of each segment type:

- V and C alleles: -penalty -5
- D and J alleles: -penalty -4 -task blastn-short

Only the highest scoring alignment was retained for each locus. Putative novel alleles (those without 100% identity matches in existing databases) underwent additional validation through manual inspection of raw read alignments in IGV to verify that the new polymorphisms were not heterogenous or located in homopolymer regions.

Custom BLAST databases were constructed using deduplicated *IGH* and *IGL* V, D, and J alleles extracted from our assembly using SeqKit v2.3 for deduplication (Shen et al. 2016). We supplemented these with the constant genes and with reference *IGK* alleles from IMGT GENE-DB due to challenges in fully resolving the *IGK* locus in the assembly.

For methylation analysis, reads were mapped to the assembled contigs and filtered for alignments with >80% coverage and >95% identity.

Methods S3: Long Read Single-cell Transcriptome Sequencing

Amplification reactions consisted of 50 µL LongAmp Taq 2x Master Mix (New England Biolabs), 1 µL 10 µM forward primer, 1 µL 10 µM reverse primer and 4 ng cDNA input in a final

volume of 100 µL. Thermal cycling was performed using 30 seconds at 95°C for initial denaturation, followed by 14 cycles of 15 seconds at 95°C for denaturation, 15 seconds at 60°C as annealing, 6 minutes at 65°C for extension, and final extension at 65°C for 10 minutes. PCR products were purified using 0.7x v/v AMPure XP (Beckman Coulter) with 80% ethanol washes and eluted into 10 µL of water for downstream processing. DNA was quantified using the Qubit fluorometer (Thermo Fisher Scientific).

Methods S4: IG Transcript-enriched Transcriptome Sequencing

The post-capture PCR of the sample-bead conjugate was performed with 25 µL LongAmp Taq 2x Hot Start Master Mix (New England Biolabs, M0533), 1 µL 10 µM forward (10x_cDNA_fwd, read 1) primer, and 1 µL 10 µM reverse (10x_cDNA_rev, TSO) primer (Supplemental Table S5) following the long amplification PCR protocol. Thermal cycling was performed using 30 seconds at 95°C for initial denaturation, followed by 14 cycles of 15 seconds at 95°C for denaturation, 15 seconds at 60°C as annealing, 6 minutes at 65°C for extension, and final extension at 65°C for 10 minutes. PCR products were purified using 1.5x v/v AMPure XP (Beckman Coulter) with 80% ethanol washes and eluted into 25 µL of water for downstream processing. DNA was quantified using the Qubit fluorometer (Thermo Fisher Scientific).

Methods S5: Expression Analysis

Raw sequencing data was processed using two parallel workflows. Illumina reads were processed through Cellranger (10x Genomics) using default parameters. Oxford Nanopore reads were processed using wf-single-cell (https://github.com/epi2me-labs/wf-single-cell).

For Seurat v5 (Hao et al. 2024) processing, initial quality thresholds were:

- Illumina MBC data: min.cells = 3, min.features = 200
- Oxford Nanopore ASC data: min.cells = 3, min.features = 100
- Oxford Nanopore transcript data: min.cells = 3, min.features = 200

Additional filtering was applied to retain cell barcodes with:

- Illumina and Oxford Nanopore gene expression: nFeature_RNA between 1500-9000
- Oxford Nanopore transcript expression: nFeature_RNA between 200-7500

All matrices underwent final processing using Seurat's NormalizeData function with scale.factor = 10000 and subsequent log transformation.

Methods S6: Antibody Synthesis

Batch 1 - Full IgG1 antibodies: 45 unique heavy/light chain pairs were randomly selected from 214 ASC-gated cells. The CRO GenScript synthesized and cloned sequences into pcDNA3.4 vectors containing:

- Heavy chain: human G1 constant region

- Kappa chain: human kappa constant region
- Lambda chain: human lambda constant 2 region

Vectors included signal sequences and kozak sites. After co-transfection into Expi293 cells, supernatants were collected at 7 days (GenScript) and quantified by BLI (Octet Red 96, Sartorius) using protein A biosensors against IgG1 isotype control antibody standard.

Batch 2 - ScFv-Fc fusions: From the remaining ASC cohort, 50 unique sequences were selected and heavy/light chain variable sequences were synthesized by Twist Biosciences as single chain variable fragments with G_4S^3 linkers and cloned into pFuse-hG1-Fc2 vectors (InVivoGen, San Diego, CA) between Il-2 signal sequence and IgG1 Fc tag (Hinge+CH2+CH3). 3 µg of vector DNA was transfected into $7.5x10^6$ Expi293 cells using Expifectamine (Thermo Fisher Scientific) and grown in serum-free media (3 mL/well). Supernatants were collected at 5 days and quantified by BLI.

Methods S7: Cell Surface Binding Assays

Antibody binding to cell surface expressed mumps and measles envelope glycoproteins were performed two different ways. For initial screening, pCAGG expression plasmids containing MuV-F, MuV-RBP/HN, MeV-F, and MeV-RBP/H under the CMV enhancer and chicken beta actin promoter were independently transfected into Expi293 cells and then processed for FACS analysis 48 hours post-transfection. Cells were washed 3x in FACS Buffer (1X PBS, 0.5% BSA, 2 mM EDTA) and resuspended at a density of 1 x 10^6 cells/ml plated (50 µL) in individual wells of a 96 well plate. Transfected supernatants from the synthesized recombinant monoclonal antibodies were then added 1:1 to the cells and stained for 1 hour on ice. Cells were then washed 3x in FACS Buffer and stained on ice for 1 hour with goat anti-Human IgG Fc Alexa Fluor™ 647 secondary antibody (Thermo Fisher Scientific), protected from light. Cells were washed again 3x and resuspended in 200 µL. Binding was read by flow cytometry on an IntelliCyte High Throughput Flow Cytometry (IntelliCyte/Sartorius).

For confirmation of putative positive MeV binders, antibodies were used to detect MeV envelope glycoproteins (F or RBP/H) expressed on infected Raji-DC-SIGN B cells. Briefly, RAji-DC-SIGN cells were infected at an MOI of 0.01 and after 3-5 days of infection, the cells were seeded at 5 × 10^4 cells per well in U-bottom 96-well plates (Fisher Scientific; #163320). Two dilutions of antibody, 10 µg or 1 µg and 1:300 or 1:3000 dilution of control MMR-vaccinated sera were made up to a total volume of 100 µL and incubated with infected cells for 2 hours at 4°C, then washed twice with FACS Buffer (1% BSA, 0.1% Sodium Azide, and 1 mM EDTA in PBS). After the 2nd wash, 1:2000 dilution of secondary Alexa FLUOR-647 GOAT-Anti human IgG [H+L] (Invitrogen; #A-21445) was added to the cells for 30 minutes followed by 3 washes with FACS Buffer, and the cells were fixed for 10 minutes in 2% Formaldehyde at room temperature before detection on the Attune NxT Flow Cytometer (Thermo Fisher Scientific). FCS files were analyzed using FlowJo v10.

A similar protocol was used to assess binding of synthesized antibodies to HCMV. A HCMV-GFP reporter virus (AD169) was used to infect normal human dermal fibroblasts in a 96-well format at MOI of 0.2. At 72 hpi cells were fixed and permeabilized and then stained with 1 ug of the indicated MMR clone. The binding of the primary MMR mAb was detected by the

secondary AlexaFluor-647 goat-Anti human IgG [H+L]. Cytogam (pooled CMV + IgG) was used as a positive control. Fluorescence intensity was quantified by the Celigo image cytometer.
